# Incubation of brain organoids with fluorescent carbon nanodots

**DOI:** 10.1039/d6ra01285j

**Published:** 2026-04-08

**Authors:** Carla Sprengel, Ute Fischer, Arndt Borkhardt, Rainer Haas, Thomas Heinzel

**Affiliations:** a Condensed Matter Physics Laboratory, Heinrich-Heine-University Düsseldorf Germany Thomas.Heinzel@hhu.de; b Department of Pediatric Oncology, Hematology and Clinical Immunology, Medical Faculty, Heinrich-Heine-University Düsseldorf Germany; c German Cancer Consortium (DKTK), Partner Site Essen/Düsseldorf Düsseldorf Germany; d Center for Integrated Oncology Aachen Bonn Cologne Düsseldorf (CIO ABCD) Germany; e Department of Haematology, Oncology and Clinical Immunology, Medical Faculty, Heinrich-Heine-University Düsseldorf Germany

## Abstract

Brain organoids are exposed during their growth to fluorescent carbon nanodots (CNDs). We observe that the CNDs penetrate through the whole organoid in all maturation stages and are taken up by viable cells irrespective of their type and location, most likely by endocytosis. They remain inside the cells after enzymatic dissociation of the organoids. No influence of the CNDs on the organoids' viability after 48 h exposure during the first 15 weeks could be detected. Our results suggest that CNDs are interesting candidates for multifunctional fluorescent labeling in organoid research and development, such as structural imaging or drug screening.

## Abstract 2

Fluorescent carbon nanodots stain characteristic structures of brain organoids.

## Graphical abstract

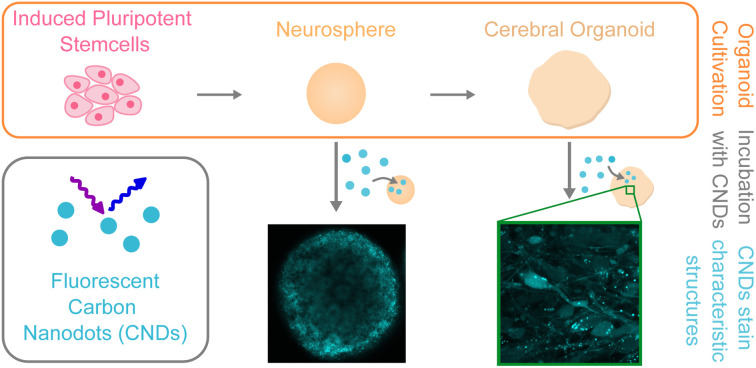

## 1 Introduction

Over the past decade, organoid research^1^ has evolved into a promising, multi-purpose field with high potential in areas of application as diverse as drug screening, animal studies replacement, personalized medicine or disease modeling.^2–4^ In many organoid based studies, nanoparticles are used as fluorophores that attach selectively to molecules of interest and/or as carriers for drug delivery. In addition to the properties these particles have to meet for applications in cellular studies, like low toxicity, high quantum yield, functionalization options and metabolic inertness, their use in organoid-based studies requires them to penetrate deeply into the sample, *i.e.*, typically through tens of cellular layers on acceptable time scales. This asks for a low-mass nanoparticle with a high diffusion coefficient, in particular with respect to the extracellular space.

In the present work, we study the suitability of fluorescent carbon nanodots (CNDs) for applications in brain organoids^5,6^ as a model system. The uptake is examined by fluorescence microscopy as well as by flow cytometry of the cells obtained from disassembled organoids. Despite some existing knowledge gaps, like the long-term toxicity and stability of nanomaterials in organoids or the transferability of results between different organoid models, as well as challenges regarding *e.g.* the lack of standardization across studies and the risk of interference of nanomaterials with biological assays and techniques (see Shi *et al.*^7^ for a recent review), previous studies on comparable systems have shown promising results. For example, graphene oxide nanoparticles are versatile fluorescent markers and carriers in organoid-based pancreatic cancer research,^8^ and they have been used for targeted gene therapy in organoid models for luminal breast cancer.^9^ Furthermore, CNDs have been demonstrated to act as nano-heaters in phototherapy applied to breast cancer organoids.^10^

The paper is organized as follows. In Section 2, the choice of the system under study is motivated, the application of the CNDs and the growth of the organoids are described, and their properties are characterized. The experimental methods are described in Section 3. In Section 4, the experimental results related to the incubation of the organoids with the CNDs and their distribution across the organoid as well as within the cells is specified, and the results are discussed. We end with conclusions and an outlook in Section 5.

## 2 Motivation for the choice of the study system

### 2.1 Carbon nanodots

Compared to the widely used II/VI – semiconductor quantum dots like CdSe – or ZnS – based particles,^11^ CNDs have a distinctly different character, see ref. 12 for a recent review. They consist typically of a graphene flake or disordered stacks of a few layers thereof. Due to carbon as their main constituent and their quasi-two-dimensional character, they have a much lower mass around 2 kDa, which is about two orders of magnitude below, for example, the frequently used CdSe/ZnS – quantum dots. Furthermore, they are intrinsically water soluble,^13,14^ which makes a hydrophilic coating obsolete. Also, due to the hydrogen termination of the CND edges,^15^ it is relatively easy to attach a variety of molecules like proteins or drugs, and they have a particular low toxicity, at least on the cellular level.^16,17^ On the other hand, the fluorescence properties of many CNDs are poorly understood: the absorption- and fluorescence spectra are frequently, but not always, incommensurate with the size quantization picture, and the fluorescence may rather originate from tightly bound small molecular fluorophores (see ref. 18 for a review). In such CND systems, the fluorescence frequency band is therefore not tunable and fixed in the blue. Taken together, CNDs represent an alternative to the established quantum dot fluorescence markers, which is complementary in many ways. Due to their small size and their low toxicity compared to other semiconductor quantum dots, they appear particularly well suited for fluorescence studies on organoids, where a large penetration length and long study time intervals after incubation are required.

Due to the strong absorption of UV and blue light in tissue and occurrence of cellular autofluorescence in the blue range, near infrared (NIR)-emissive samples show superior features for imaging in tissue, especially with emission in the NIR-II range above 1000 nm since this is the most favorable window for deep-tissue imaging and high signal-to-noise ratios (see Gil *et al.*^19^ for a review on NIR-emissive quantum dots and Parvin *et al.*^20^ for a recent review on NIR-II fluorescent probes). Even though NIR-emissive CNDs have already been established in general, there is still a lack of protocols for CNDs that show strong emission with high quantum yields in the favorable NIR-II window.^21–23^ For example, the presented NIR-II emitting CNDs for application in *in vivo* mouse models by Wang *et al.*^24^ and Guo *et al.*^25^ still show low quantum yields of 1.0% respective 0.2% in (aqueous) solution. Since the most promising NIR-II probes are still in its infancy and remains challenging *per se*, we opted for using a well-established, readily available and profoundly tested type of CND for our studies.

The uptake of CNDs in solution by adherent cells has been studied extensively over the past few years.^14,26,27^ It has been established that the uptake occurs *via* endocytosis, with the CNDs getting stored mostly,^28^ but not exclusively, in the lysosomes,^29^ the function of which appears to be unaffected by the presence of the CNDs.^30^ Furthermore, experiments have been reported where the CNDs are used as active elements in drug delivery.^30–33^ The diffusion and penetration depth of nanoparticles (NPs) into tissue is in general highly dependent on different factors, one of them being their surface composition.^34^ The CNDs used in this study present COOH/C–OH and C=O/C–O functional edge groups.^17^ The deprotonation of the carboxyl groups in aqueous solution can be associated with a negative zeta potential which may be weakened by the presence of further amine groups.^14,35^ As anionic NPs exhibit a limited interaction with the extracellular matrix in comparison to cationic NPs,^36^ we expect our CNDs to penetrate the whole organoid. When NPs enter a biological medium, the spontaneous formation of a protein corona usually occurs from proteins present in the cell culture media. This corona can mask the NPs surface and create a new biological identity influencing the interaction between NPs and biological systems.^37,38^ The formation of a protein corona can be enhanced or inhibited by additional surface coating steps, as Dridi *et al.*^39^ demonstrated. They showed that AuNPs coated with dihydrolipoic acid (presenting a carboxy group on its one end) lead to a non-specific adsorption of proteins while adding hydrophilic PEG blocks or zwitterion groups prevented the formation of a corona. Thus composition of the functional groups and surface charge of the carbon nanodots can also influence the spontaneous formation of a protein corona which may also hamper the successful penetration of the particles through the organoid.^40^ Nevertheless, the pristine CNDs analyzed in this study show high penetration rates into tissue (at least up to 250 µm) as seen in previous studies where precision-cut tissue slices of mouse liver tissue^28^ were analyzed and were chosen as promising candidates for this study.

### 2.2 Brain organoids

Organoid models are becoming increasingly important in many fields like, *e.g.*, personalized therapy, development of alternatives to animal testing or disease modeling. We opted for brain organoids as a model system due to their particularly broad range of applications,^5,41,42^ which extends beyond other organoid systems in terms of psychiatric disease modeling and brain-like artificial intelligence.^3^ Furthermore, the CNDs are known to accumulate in lysosomes, and endolysosomal dysfunction has been identified as a key mechanism in neurodegenerative diseases,^29^ in particular in relation to Alzheimer's disease,^43^ Parkinson's disease^44^ and frontotemporal dementia.^45^

**Fig. 1:**
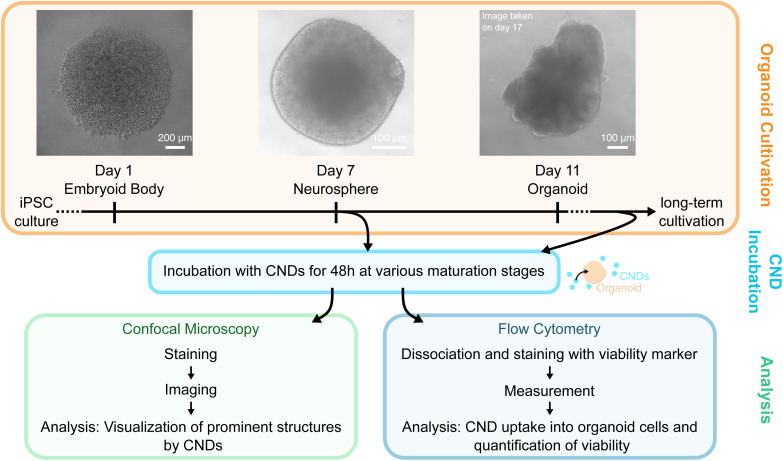
The iPSC-derived cerebral organoids were used as a model system to study the uptake of CNDs into tissue. After the embryoid bodies have reached the neurosphere (or organoid) maturation stage, they were used for CND uptake experiments. The uptake was analyzed by confocal microscopy to reveal the intra-organoid distribution and for visualization of the distinctive rosette-like structure of neurospheres at early maturation stages. Flow cytometry is used to verify the cellular CND uptake and to quantify of the organoid viability.

For the present study, we derived human cerebral organoids from induced pluripotent stem cells (iPSCs) according to the protocol described by Gebing *et al.*^46^ An overview of the organoid development is depicted in the upper part of Fig. 1. First, the iPSCs form the so-called embryoid body (EB) which will evolve into neurospheres after seven days and exhibit characteristic neural rosette structures. At day 11, the neurospheres have evolved into organoids which can be kept several weeks to months in culture.

### 2.3 Incubation of the organoids with CNDs and restrictions to the experimental processing and data acquisition

To study the uptake of CNDs into the cells of the organoids, an incubation time of 48 h and a concentration of 0.5 mg ml^−1^ was chosen as it showed a good particle uptake by cells without influencing the cellular viability in 2D cell models.^28^ Organoids at different maturation stages were used which show differences not only with respect to their composition of cell types, but also regarding their size and structural organization. The organoids were analyzed by live-cell confocal fluorescence imaging in combination with flow cytometry. Both methods are restricted by the exclusion of all procedures that include fixation and/or permeabilization steps (*e.g.* immunofluorescence staining and tissue clearing) since the CNDs would diffuse through permeabilized membranes. For the imaging of organoids, the restrictions are the penetration depth of the light into the dense organoid tissue, the working distance of the imaging setup as well as the staining for different cell types with live-cell fluorophores. Neurospheres from day 9 in culture were chosen to study the CND uptake and staining potential because of their uniformity, small size and their characteristic neural rosette patterns.^41^ For the studies of the uptake by more mature organoids, organoids at day 28 in culture were chosen. Here, the inter-organoid variability (with respect to size, shape and structure) was already strongly pronounced, reflecting the individual growth and differentiation histories.

Regarding the live-cell markers, we faced the challenge that many established staining protocols for neurons are based on Nissl- or antibody-staining which requires permeabilized membranes and is therefore not compatible with the CND detection in live-cell studies as described above. We could not establish a reproducible neuron-specific staining procedure without permeabilization and a reliable output. For astrocytes, we chose the marker sulforhodamine 101 (SR101) since it is widely used for live-cell staining of astrocytes. In rodent astrocytes, SR101 is taken up *via* a specific transporter and remains in living-cells.^47,48^ Whether human iPSC derived astrocytes take up the marker accordingly is still to be investigated.

The flow cytometry analysis was limited by the dissociation process of the organoid tissue and the staining for different cell types. To obtain a single cell suspension, an enzymatic dissociation based on papain/DNAse I treatment was chosen which provides a high cell count of viable cells, see Section 4.1 for the discussion. This method has the disadvantage that the surface (CD-) markers required for cell-specific gating are degraded by the enzyme. A non-enzymatic, mechanical dissociation, on the other hand, leads to an unacceptably low single-cell yield for early stage organoids and insufficient low viability.

## 3 Experimental

### 3.1 Carbon nanodot synthesis and characterization

The preparation and characterization of the CNDs used in the present study has been reported elsewhere in great detail,^17^ and we therefore restrict ourselves to the most relevant information. We have synthesized the CNDs following a modified version of the process reported by Qu *et al.*,^13^ where 210 mg anhydrous citric acid (Thermo Fisher, #036664.22) and 340 mg diethylenetriamine (DETA, Sigma-Aldrich, #8032740100) are stirred for 5 min at room temperature. The solution is heated afterwards in a sealed vessel by microwave irradiation under continuous stirring at 180 °C for 2:30 min in a microwave furnace (CEM Discover). The product was dissolved in 10 ml deionized water and dialyzed (Repligen, Float-A-Lyzer, 0.1–0.5 kD, #G235061) against 2 l deionized water for 48 h with three water exchanges. After dialysis, the remains were lyophilized, the mass was determined and the CNDs were dissolved in Dulbecco's phosphate-buffered saline (DPBS) for cellular studies. For incubation with the organoids, the CNDs were sterile filtered and diluted to the required concentration in organoid medium.

The characterization measurements of our CNDs have been described in great detail elsewhere.^17^ They are composed of approximately 40% C, 33% O, 19% N and 8% H atoms. X-ray photoelectron spectroscopy (XPS) shows that 29% of the carbon bonds consist of C–C single bonds, with the rest equally distributed among C–O and C–N bonds. The average lateral size of the CNDs was measured *via* high-resolution transmission electron microscopy to 3.3 nm with a Full Width at Half Maximum (FWHM) of 0.6 nm. Atomic force microscopy studies reveal CND heights between 1 nm and 2 nm, which corresponds to two to three graphene layers per CND.

The spectroscopic studies show an absorption band that peaks at 240 nm and 350 nm with a long wavelength tail that extends into the visible. These two components have been assigned to π–π* transitions of C=C bonds and to n–π* transitions of C=O bonds, respectively.^13^ Excitation between 320 nm and 400 nm causes a fluorescence emission with a maximum close 460 nm and a FWHM of approximately 50 nm. A quantum yield of 23% for excitation at 360 nm was determined.

Absorption and fluorescence spectra, the quantum yield as well as ^1^H NMR- and FTIR-Spectra of the CNDs used in this study are displayed in Fig. S1 and S2 of the SI. The absorption and fluorescence spectra were obtained with a Horiba Duetta™ Fluorescence and Absorbance Spectrometer was used. ^1^H NMR measurements were recorded with a Bruker Avance III – 600 or Bruker Avance NEO evo 600 by the CeMSA@HHU. The data was then processed and displayed with MestReNova (14.2.0-26256). FTIR spectra were obtained with a Bruker Tensor 37 spectrometer.

### 3.2 Generation of human cerebral organoids

#### 3.2.1 Maintenance of iPSCs

For the culture of the iPSCs (the human HW8 cell line has been supplied by the Zhengping Zhuang Lab, National Cancer Institute, NIH, see also Jepsen *et al.*^49^ for further information), dishes were coated with Geltrex® (Gibco, #A1413302) according to the manufacturer's instruction (thin-layer method). The iPSCs were kept in mTeSR Plus medium (STEMCELL Technologies, #100-0276) with medium exchanges every other day, and gently passaged with a Versene solution (Gibco, #15040066).

#### 3.2.2 Growth of the embryoid bodies

The human cerebral organoids were grown by following the protocol of Gebing *et al.*,^46^ which was previously adapted from Gabriel and Gopalakrishnan.^41^ The iPSCs were gently washed with DMEM/F-12 (Gibco, #31330095) and incubated with Accutase (Merck, #A6964) for 5–7 min at 37 °C to form a single cell suspension. The suspension was diluted in mTeSR Plus medium and centrifuged at 500 g for 4 min. The supernatant was discarded afterwards. The cells were resuspended in Neural Induction Medium (NIM, STEMCELL Technologies, #05835) supplemented with 10 µM Y-27632 (Selleckchem, #S1049) to yield a final concentration of 3.5 × 10^5^ cells per ml. 100 µl of the cell suspension (=35 000 cells) were seeded in each well of an ultra-low attachment U-bottom 96-well plate (Corning, #7007) and centrifuged at 500 g for 3 min to form the EB aggregates. The EBs were incubated at 37 °C and 5% CO_2_ in humidified air. This step marks the day 1 of the organoid culture. On day 3, 100 µl NIM was added to each well. On day 5, 100 µl medium was removed from each well and 100 µl fresh medium was added.

#### 3.2.3 Neurosphere cultivation

On day 7, the now so-called neurospheres were collected with a cut 1000 µl-pipette tip. The neurospheres were then transferred into 24-well plates with 2 neurospheres per well in 500 µl neurosphere medium and incubated at 37 °C and 5% CO_2_ in humidified air. The neurosphere medium consists of 1 : 1 (v/v) DMEM/F-12 (Gibco, #31330095) and Neurobasal™-A medium (Gibco, #10888022) supplemented with 1 : 100 (v/v) N2-Supplement 100× (Gibco, #17502048), 1 : 50 (v/v) B27-Supplement w/o Vit.-A 50× (Gibco, #12587010), 300 mg per l l-glutamine (Merck, #G7513), 100 U per ml penicillin and 100 µg per ml streptomycin (Merck, #P0781), 1 : 100 (v/v) MEM NEAA 100× (Gibco, #11140035), 130 µg per ml insulin (Merck, #I9278) and 55 µM β-mercaptoethanol (Gibco, #21985023). On day 9, 500 µl of medium was added to each well.

#### 3.2.4 Organoid cultivation

On day 11, the organoids were transferred into a spinner flask (Pfeiffer, #182026) which was stirred at 25 rpm and contained 1–2 ml medium per organoid. The organoid medium consists of the neurosphere medium supplemented with 5 µM SB431542 (Selleckchem, #S1067) and 0.5 µM dorsomorphin (Selleckchem, #S7840). The organoids were incubated at 37 °C and 5% CO_2_ in humidified air and half of the medium was replaced with fresh medium every 4–6 days.

### 3.3 Incubation with CNDs

For cell experiments, organoids of a certain age were transferred to 6-well plates for the incubation with CNDs. Depending on the size and age of the organoids, one to three organoids were incubated per well in 3–4 ml of medium. Neurospheres were incubated according to the protocol at that time point. To yield the final concentration of 0.5 mg ml^−1^, the corresponding amount of CNDs solved in DPBS (20 mg ml^−1^) was added to the wells. An equal amount of DPBS was added as a solvent control to the control samples. The organoids were then incubated on an orbital shaker, and neurospheres were incubated at rest at 37 °C and 5% CO_2_ in humidified air for 48 hours.

### 3.4 Flow cytometry

For the enzymatic dissociation, reagents of the Papain Dissociation System (Worthington, #LK003160) were used with adjustments of the manual. The papain and DNAse I vials of the kit were dissolved in DMEM/F-12 and reconstituted following the instructions of the manual to yield the enzyme solution. After washing the organoids with DPBS, they were incubated in the enzyme solution for 30 to 45 min at 37 °C and 5% CO_2_ with constant agitation. Every 15 minutes, the organoids were gently triturated by slowly pipetting each organoid a few times using 1000 µl and 100 µl pipette tips. The final cell suspension was filtered using a 40 µm cell strainer to yield a single-cell suspension and to filter out remaining aggregates. DMEM/F-12 with 10% Fetal Bovine Serum (FBS, Sigma, #F2442) was added to stop the enzymatic reaction. The suspension was centrifuged at 300 g for 5 min and the supernatant was discarded. The cells were resuspended in FACS buffer (DPBS with 0.5% BSA (Roth, #1ETA.2) and 0.5 mM EDTA (Roth, #9152.1)) and filtered again using 40 µm cell strainer. The mechanical dissociation was carried out analogously, but without the incubation step in the enzyme solution. It was performed by pipetting the organoids using 1000 µl and 100 µl tips until a suspension with single cells and as few cell aggregates as possible was reached. The suspension was filtered with a 40 µm cell strainer to get rid of larger cell clusters and further processed as described above for the enzymatic method. For staining with the JC-1 dye (MedChemExpress, #HY-15534) and FCCP (MedChemExpress, #HY-100410) incubation, after the described dissociation, the cells were re-suspended in organoid medium and 100 µM FCCP was added only to the desired samples. After an incubation for 15 min at 37 °C on an orbital shaker, 2.5 µM JC-1 was added to the samples and incubated for 20 min. Afterwards, the cell suspensions were washed twice with DPBS and centrifuged at 300 g for 5 min. The stained samples were then resuspended in FACS buffer and filtered again with a 40 µm cell strainer. A concentration of 0.5 µg per ml 7AAD (7-Aminoactinomycin D, BioLegend, #420404) was added prior to the measurement, with an incubation time of at least 5 min in the dark at room temperature, without a washing step.

The data acquisition was conducted using a CytoFLEX cytometer (Beckman Coulter). The following setup was used: (1) CNDs: PB450 channel (ex. 405 nm, em. 450 nm/45 nm BP), (2) 7AAD: PC5.5 channel (ex. 488 nm, em. 690 nm/50 nm BP), (3) JC-1 monomers: FITC channel (ex. 488 nm, em. 525 nm/40 nm BP), (4) JC-1 aggregates: PE channel (ex. 488 nm, em. 585 nm/42 nm BP). The data analysis was performed using FlowJo™ v10.10.0 software (BD Life Sciences). Compensation for JC-1 was set manually to PE–FITC: 25% and FITC–PE: 12%. For the statistical analysis, OriginPro 2021b (OriginLab Corporation) was used.

### 3.5 Confocal fluorescence microscopy

Organoids/neurospheres were carefully washed 2–3 times with DPBS and incubated for 30 min in medium containing 10 µM sulforhodamine 101 (“SR101”, MCE, #HY-101878). For lysosomal staining, 100 nM LysoTracker™ Deep Red (“LysoTracker”, Invitrogen, #L12492) was added to the staining medium. The incubation was extended to 90 min. The organoids/neurospheres were washed 2–3 times with DPBS afterwards and transferred to imaging dishes (ibidi, #81817) with phenol red-free 1 : 1 (v/v) DMEM/F-12 and neurobasal medium.

For imaging, a Zeiss LSM710 confocal fluorescence microscope, equipped with an EC Plan-Neofluar 10×/0.30 M27 objective and a Plan-Apochromat 63×/1.40 Oil DIC M27 objective, was used. The lasers and detectors were set to (i) CNDs: ex. 405 nm, em. 440–481 nm, (ii) SR101: ex. 543 nm, em. 575–616 nm, (iii) LT: ex. 633 nm, em. 638–747 nm. Since the settings were kept constant, qualitative intensity comparisons between the images obtained with the same magnification are possible. Processing, analysis and plotting of the images was performed using FIJI^50^ (ImageJ v1.54f) and Omero.figure (v7.2.1).

## 4 Results

This section is structured according to the main findings of the study.

### 4.1 Mechanical dissociation of the organoids leads to low viability compared to enzymatic dissociation

In order to study the effect of the dissociation protocol on the cellular viability, we chose (see also Section 2.3) (i) an enzymatic dissociation protocol based on papain, and (ii) a purely mechanical dissociation. 15 weeks old organoids were chosen for this study, as they are mature by this time point and reached sizes up to a few mm. They were processed as described in the methods section. 7AAD was added to the obtained single cell suspension prior to the measurement as a viability marker. 7AAD binds to the G–C base regions in the DNA of dead cells after diffusing through the porous cell membranes and is therefore widely used as a live/dead (7AAD^−^/7AAD^+^)-marker.^51^ The viability was determined as the percentage of remaining cells after 7AAD^+^ exclusion. It should be mentioned that the measured viability corresponds to the cellular membrane integrity and not to the viability obtained from *e.g.* MTT or XTT assays reflecting the metabolic activity. A JC-1 staining was performed to analyze the mitochondrial function of the cells and is discussed below.

**Fig. 2:**
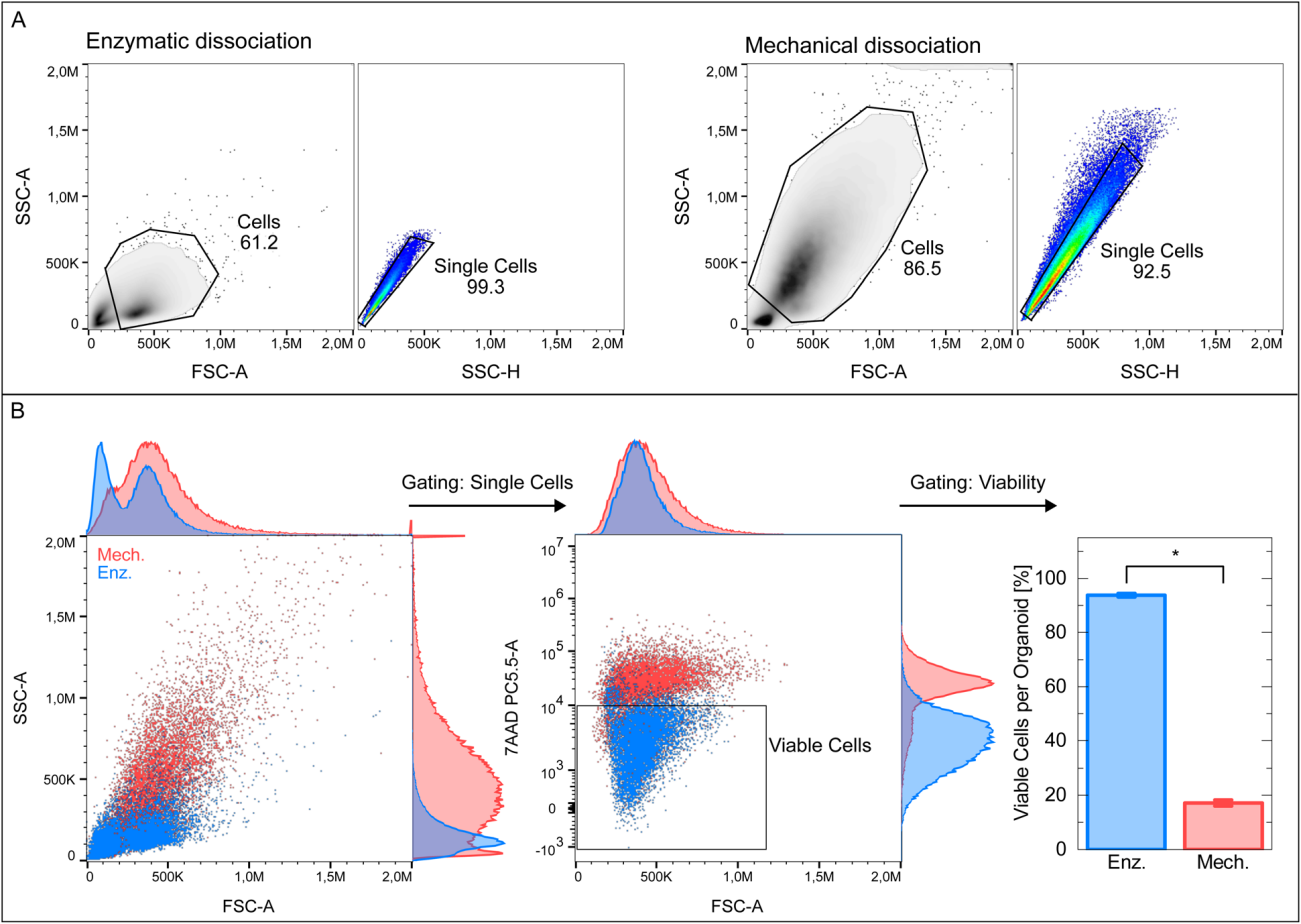
Flow cytometry analysis of 15 weeks old organoids after enzymatic or mechanical dissociation. Enzymatic dissociation was performed using papain and DNAse I, while the mechanical dissociation was done only by mechanical trituration using pipette tips. (A) SSC:FSC and SSC-A:SSC-H scatter plots of the organoids that were dissociated enzymatically or mechanically. (B) Comparisons of the scatter plots and of the cellular viability after enzymatic or mechanical dissociation. Cells were stained with 7AAD as a viability marker. Viable cells were determined by 7AAD^+^ exclusion. 4 pooled organoids were measured in 4 technical replicates per sample. An independent two-sample *t*-test with Welch's correction was performed with a significance level of *α* = 0.05: *p < 0.05 (*p* = 4.9 × 10^−10^).

In Fig. 2A, exemplary gating schemes to obtain the single cell populations are depicted for the two samples. As can be seen in Fig. 2B, the used dissociation method has a major impact on the cellular granularity and viability. It is observed that the SSC and FSC signals of mechanically dissociated organoids (MDOs) reach higher intensities and show broader intensity distributions than the signals of enzymatically dissociated organoids (EDOs). This indicates that the cells of the MDOs show more diverse size and granularity distributions. In the FSC:7AAD scatter plots, the higher amount of 7AAD binding to the MDO cells in comparison to the EDO cells is visible. Gating for viable cells based on the 7AAD^+^ exclusion leads to viability rates of 94% for the EDOs and 17% for the MDOs. A viability of 17% would not be sufficient for most experiments. As we considered a viability of less than 17% as far from being satisfactory, we used the EDOs for our CND uptake experiments.

Additionally, JC-1 staining was performed with 8 week old organoids to analyze the effects of the dissociation method on the mitochondrial membrane potential which can be taken as a measure for the metabolic function. The results are presented in Fig. S5 and SI Table 1. The mechanical dissociation leads to a statistically significant and biologically relevant decrease of the metabolic function in comparison to the enzymatic dissociation, which is in accordance with the findings from the 7AAD staining described above.

### 4.2 Viable cells take up CNDs

We begin our CND studies by analyzing the uptake of CNDs into the cells of the organoids based on flow cytometry. After the 48 h incubation with CNDs (or without CNDs under identical conditions as a control), the organoids were enzymatically dissociated using papain and DNAse I. 7AAD was added to the single cell suspensions directly prior to the flow cytometry data acquisition.

**Fig. 3:**
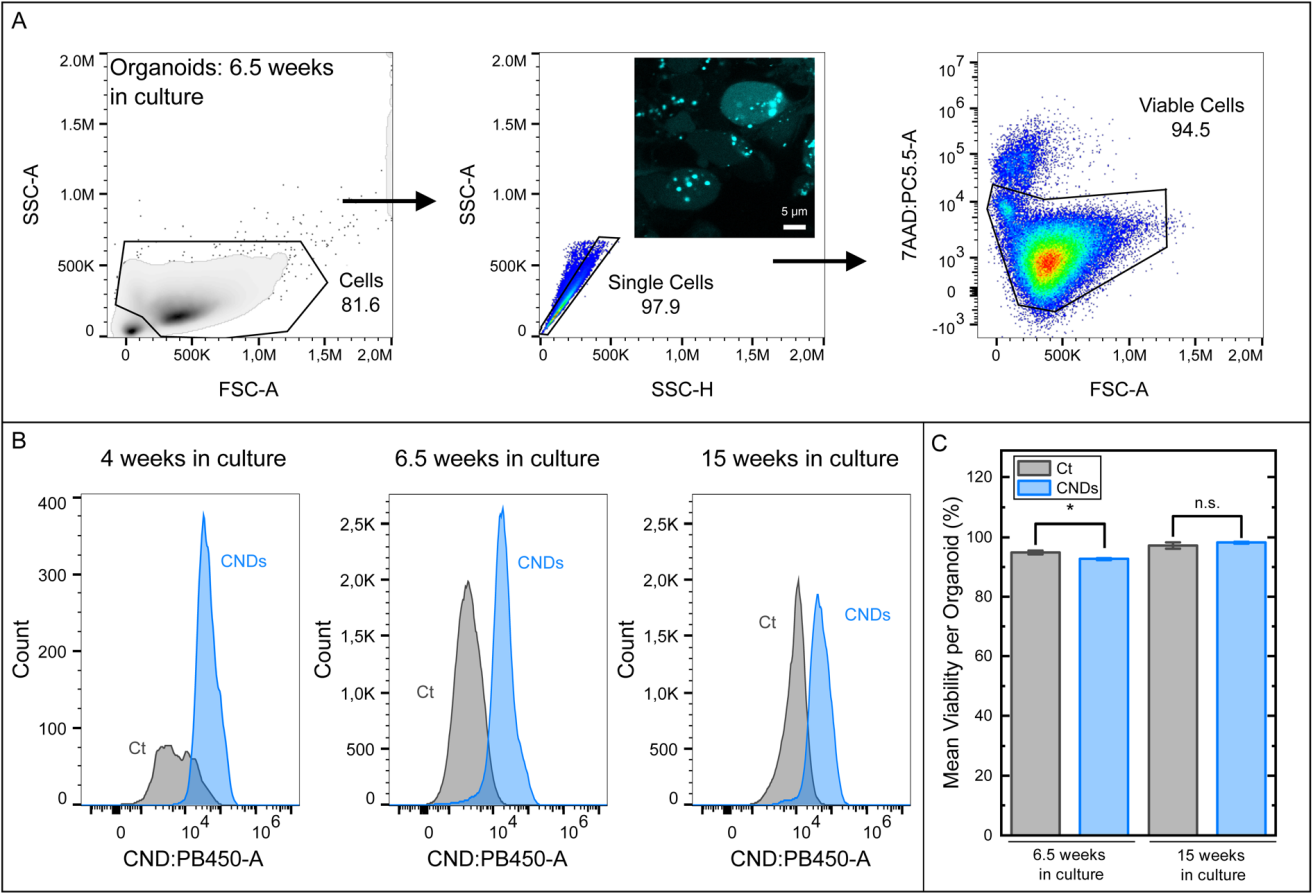
Flow cytometry analysis of the organoids after incubation with CNDs. (A) The gating strategy for the flow cytometry data analysis is displayed exemplarily. First, the single-cell population was gated by the SSC:FSC scatter plot followed by the exclusion of duplicates by the SSC-A:SSC-H plot. Viable cells were gated by 7AAD^+^ exclusion. The numbers displayed in the scatter plots refer to the percentage of the gated population in comparison to all events in that plot. Inset: the microscopy image shows CNDs inside cellular soma. (B) In the histograms, the distribution of the fluorescence signals in the CND channel are displayed for samples stained with CNDs (blue, “CNDs”) and without (grey, “Ct”) at three different growth stages of the organoids. For the 4 weeks old organoids, 3 organoids were pooled and measured in 3 biological replicates. The cell yield was too low to further determine the viability with a sufficient statistic in (C). (C) Mean viability per organoid after CND uptake in comparison to the control organoids for two different organoid ages (6.5 weeks and 15 weeks). The viability is defined as the percentage of viable (7AAD-negative) cells per sample. For all samples, the viability remains above 92%. For the 6.5 weeks old organoids, five organoids were pooled and measured in three technical replicates per condition. For the organoids of 15 weeks age, two biological replicates of 2–3 pooled organoids were measured. An independent two-sample *t*-test with Welch's correction was performed with a significance level of *α* = 0.05 (comparisons only between comparable organoids of the same age): *p < 0.05, n.s. = not significant (6.5 weeks in culture: *p* = 0.012, 15 weeks in culture: *p* = 0.10).

To select the viable cell populations, the flow cytometry data was gated as depicted in Fig. 3A. The CND fluorescence intensity distribution of the viable cells is reproduced in Fig. 3B in comparison with the organoid-derived cells without CND exposure for three different ages. For all organoids, an increase of the fluorescence intensity due to the CND exposure is observed in the blue PB450/CND channel at the expense of the pristine distribution peak (see Ct samples), which has vanished. This proves that viable cells in the organoids take up CNDs, regardless of their position in the organoid. Fluorescence signals of unstained control organoids originate from the cellular autofluorescence. It should be noted that due to the large size of the older organoids, the cells located in the inner core may show signs of necrosis, a consequence of hypoxia and lack of nutrients,^5^ and may therefore have been gated out as non-viable cells. Since the diameters of the organoids reach up to a (few) mm in the mature organoids, an exact penetration depth of the CNDs into the organoids could not be determined, but it can be estimated to exceed a value of 500 µm corresponding to about 30 cell diameters.

### 4.3 The effect of a CND incubation on the cellular viability inside organoids is small compared to other factors

The influence of the CNDs on the cellular viability in organoids of different age was studied as well. In Fig. 3C, the fractions of viable cells per organoid as obtained from the 7AAD staining are displayed. Apparently, the CNDs lead to a significant albeit very small decrease (from 94.8% to 92.6%) in our 6.5 weeks old organoids. Since previous studies have shown that cell lines in 2D culture tolerate CND concentrations up to 0.5 mg ml^−1^ over 48 h well without a significant loss of viability,^28^ we attribute this statistically significant differences to organoid-to-organoid variations and/or to handling of the organoids during the preparative processing for the measurement, rather than to a cytotoxic effect. This interpretation is also supported by the conserved viability in our organoids after 15 weeks of cultivation. JC-1 staining for determining the metabolic activity in 8 week old organoids did not show significant effects of the CND incubation either, see Fig. S5 and SI Table 1. In comparison to the influence, the mechanical dissociation has on the cellular viability as discussed above, the CNDs' influence remains marginal. It should be noted that in general, non-viable cells in organoids can originate from necrosis of the inner core as described above.

### 4.4 CNDs stain characteristic structures in neurospheres

**Fig. 4:**
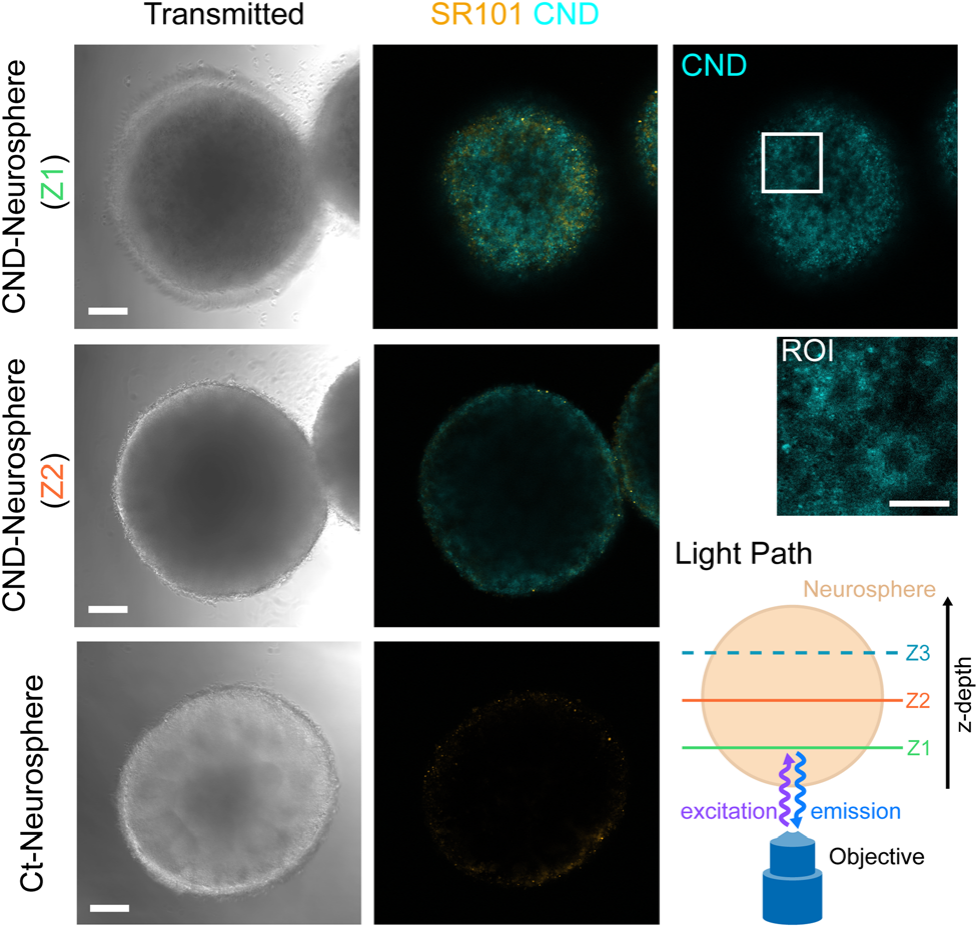
Confocal microscopy images of neurospheres at day 9 in culture. SR101 was used to stain already developed astrocytes. Images of two confocal planes of the neurosphere that was incubated with CNDs (rows “CND-Neurosphere”) are depicted. Due to the CND uptake, the characteristic neural rosettes are visualized (see also the ROI). Deeper light penetration depths (see *e.g.* the comparison of the cross sections at depth *z*1 and *z*2, lower right scheme) lead to a decreased detection of the fluorescence signal in all channels due to light scattering and absorption by the dense organoid tissue. Comparison of the CND-neurosphere with the control “Ct-Neurosphere” (bottom row) shows that the autofluorescence of the cells in the CND channel is negligible. Scale bar: 100 µm, ROI: 50 µm.

To study the staining capability of CNDs in organoids, we begin with 9 days old neurospheres which are known to form a characteristic neural rosette structure. The neurospheres were imaged after a 48 h incubation with, as well as without, CNDs (“CND-” and “Ct-Neurosphere”, respectively) using a confocal fluorescence microscope. Images of different neurosphere planes are reproduced in Fig. 4. The autofluorescence signal of the cells can be neglected in comparison to the CND signal, seen as the absence of significant intensities in the CND channel of the control neurosphere (lowermost row in Fig. 4). For a low *z*-depth (plane *z*1), a strong CND fluorescence intensity across the whole cross section is observed. The neural rosettes are identified by the spherical alignment of the stained cells with a dark lumen in their center (ROI in Fig. 4). They can be clearly distinguished from the dark surrounding as well as from the more differentiated outer layers. At this maturation stage, sulforhodamin 101 (SR101) does not stain these structures yet, due to the absence of differentiated cells. For images along deeper slices like plane *z*2, a marked intensity decay from the outside to the inside of the organoid is observed, which we attribute to the longer paths of the excitation light as well as the fluorescence light through the organoid. We were unable to detect fluorescence intensity emerging from plane *z*3, symmetric to plane *z*1 about plane *z*2 (see Fig. S6 for images from plane *z*3). This proves that the observed intensity decrease towards the inner regions is mainly dependent on the penetration depth of the exciting and/or the fluorescence light, rather than by that one of the CNDs, in the organoid. In the latter case, the fluorescence image of plane *z*3 should be identical to that one of plane *z*1. Furthermore, the autofluorescence of pristine organoids shows a similar intensity distribution. These observations are in tune with the assumption, based on the dissolved cell ensemble analysis above, that the CNDs have a penetration length of at least the organoid radius.

### 4.5 In mature organoids, the CNDs stain the neural network and cellular interconnections with high resolution

**Fig. 5:**
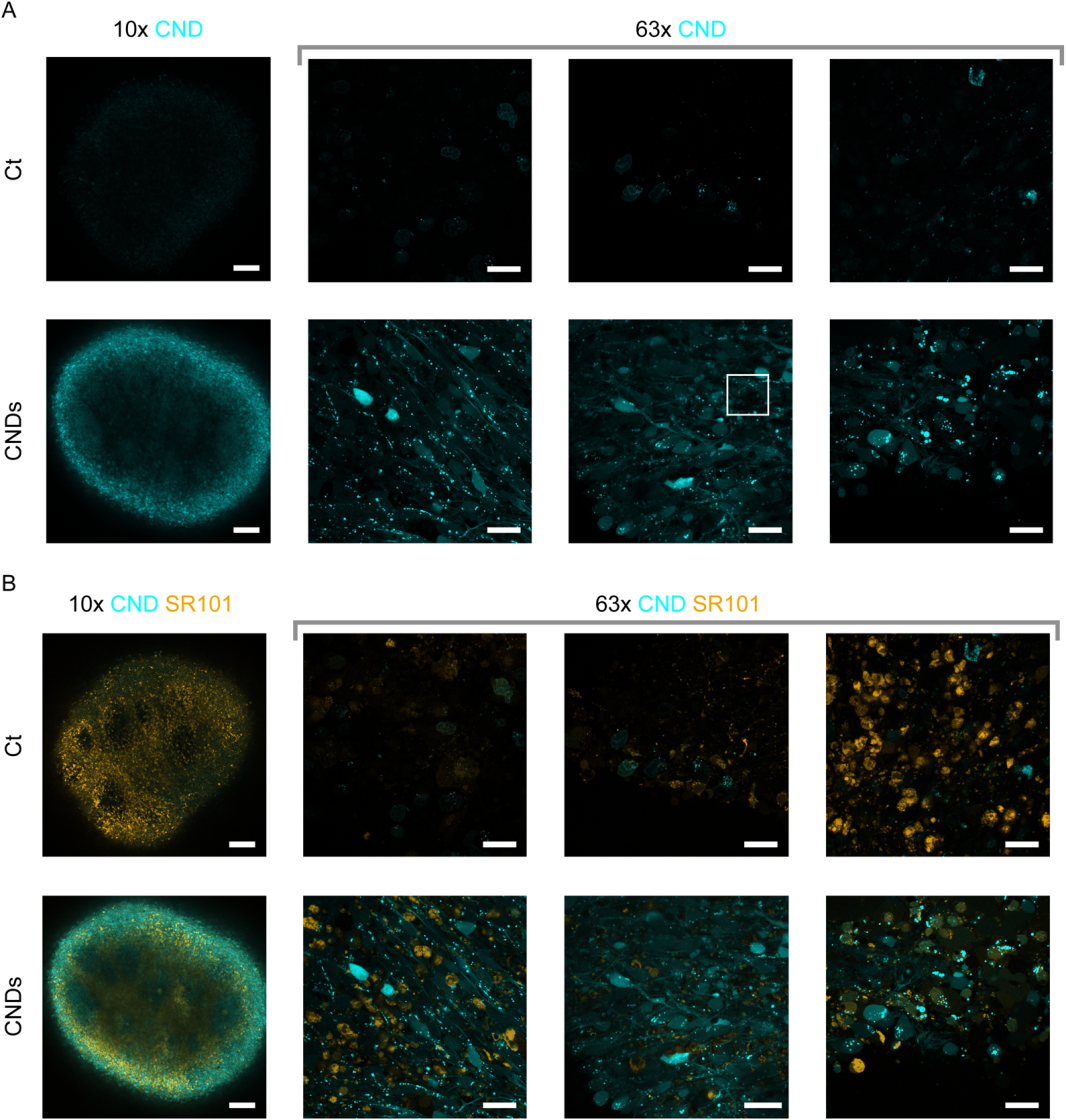
Confocal microscopy images of organoids at day 28 of culture after CND incubation (“CNDs”) or without (“Ct”). Images were obtained using the 10× or 63× magnification. In (A) the signals obtained in the CND channel are depicted for multiple replicates. The zoom of the marked region is displayed in Fig. 7. Additionally to the CNDs, the organoids were stained with SR101. The overlays of the CND and SR101 images are depicted in (B). Each image (except CT, 63× image 2 and 3) corresponds to a different organoid. Images with the 63× magnification were obtained such that the outermost regions of the organoids with the smallest loss of intensity were scanned to enable qualitative intensity comparisons. Scale bar: 10×: 100 µm, 63×: 20 µm.

In Fig. 5 images of 28 days old organoids, incubated with or without CNDs for 48 h, are depicted. The CNDs cause strong fluorescence in the blue channel compared to the control organoids (“Ct”) where the autofluorescence is weak (see Fig. 5A). As seen in the 10× magnification images (see also SI Fig. S7 for *z*-stack images), the detected CND fluorescence intensity is attenuated as the imaging depth increases. An intensity decrease is observed towards the inner regions of the organoid, as already discussed in Section 4.4 for neurospheres. A quantitative analysis of the intensity attenuation is presented in the SI Fig. S8. Similar intensity decays across the different imaging depths are observed for the CND fluorescence of stained organoids (half-value depth of 34.5 µm ± 2.7 µm) and the autofluorescence of the Ct-organoids (half-value depth of 36.0 µm ± 3.5 µm). We conclude that the attenuation is dominated by interactions of the excitation and emission light with the tissue and not by a finite penetration depth of the CNDs, which is thus large compared to the size of a typical organoid. This is consistent with the results obtained from the flow cytometry measurements, showing that the viable cells from the organoid take up organoids independently of their origin inside the organoid.

**Fig. 6:**
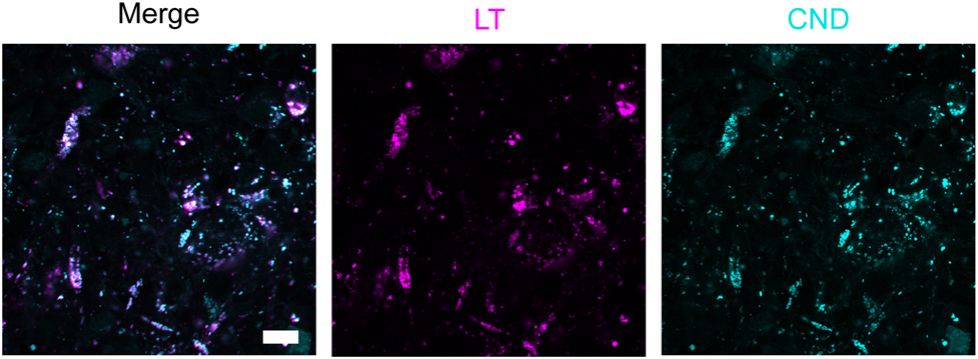
Confocal fluorescent images of an representative organoid at day 28 of culture after CND incubation. Lysosomes were stained with LysoTracker (“LT”) and are displayed in magenta. The CND channel is shown in cyan. In the merged image, overlays of CNDs and LysoTracker signals can be observed, indicating that CNDs accumulate inside the lysosomes. Scale bar: 10 µm. See also Fig. S10 for additional images.

On the cellular level (63× magnification), the typical staining pattern of CNDs is observed: CNDs accumulate in cellular structures, as seen by points of high intensity. Earlier studies suggest that these structures are defined as organelles in the endo-lysosomal pathway.^28^ A co-staining with LysoTracker, as displayed for a representative organoid in Fig. 6 was performed to identify whether the CNDs accumulate predominantly in the lysosomes. Merging of the cyan CND channel and the magenta LysoTracker channel reveals bright spots of mixed-color arising from colocalization of CNDs and lysosomes. Due to the sequential scanning mode, an acquisition delay between the two fluorescent channels occurs. As imaging of living cells was conducted, motion of lysosomes and vesicles prevents the observation of exact colocalization between those two channels. Nevertheless, the overlap was quantified using the Pearson correlation coefficient (PCC) and the Manders' overlap coefficient (MOC) for the LysoTracker signal that coincides with CND fluorescence, indicating the lysosomal filling with CNDs. For 6 different images from 3 different organoids, the mean PCC ± standard deviation (SD) of 0.33 ± 0.15 and the mean MOC ± SD of 0.53 ± 0.21 are indicating a modest colocalization. We conclude that the CNDs accumulate at least partially inside lysosomes as more than half of the LysoTracker-positive regions contain CND signals. Besides the acquisition delay discussed above, newly formed lysosomes that have not taken up either CNDs or LysoTracker yet, will also influence the PCC and MOC negatively. Since endocytosis is a primary route for transporting cargo from the extracellular space into the lysosomes,^52^ the accumulation of CNDs inside the lysosomes suggests that the CNDs are (at least partially) taken up *via* the endo-lysosomal pathway. This is in accordance with the literature, suggesting that the uptake of NPs rather relies on endocytosis than other mechanisms such as passive diffusion.^53,54^ Here, endocytosis is used as a collective term encompassing the different mechanisms usually assigned to endocytic uptake such as receptor-mediated endocytosis, receptor-independent endocytosis or macropinocytosis.^55^ The suppression of endocytosis at 4 °C reveals that the uptake of our CNDs into MCF-7 cells is negligible compared to MCF-7 cells that were incubated with CNDs at 37 °C (see SI Fig. S9). This suggests, that endocytosis is the primary uptake mechanism compared to other processes such as diffusion.

Additionally, a less intense CND signal is evenly distributed across the whole soma including dendritic/outgrowing structures. Therefore the neural network and cellular interconnections can be visualized by the CNDs. These findings are in agreement with previous observations by Hivare *et al.*^56^ where the endocytotic uptake of carbon nanoparticles into neuroblastoma derived neurons was shown and studies by Zhou *et al.*^57^ where the active transport and distribution of CNDs across (rat) neural cells and exocytosis of CNDs was detected. We note that in some images (*e.g.* bottom right-hand side in Fig. 5A), fluorescent regions with high intensities arise with a morphology (big circular dots) incommensurate with the signals expected from lysosomes or endosomes.

**Fig. 7:**
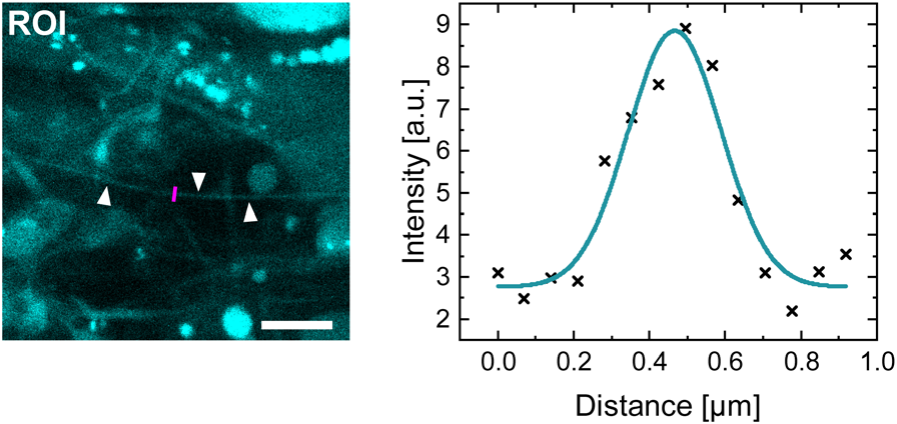
The ROI shows a zoom of the marked region in Fig. 5A for a better visualization of the staining capability of the CNDs (intensities are enhanced compared to the full image). A cellular, fibrilic outgrowth which expands over a few 10 s of micrometers is marked with arrows. An exemplary line plot along the magenta line in the ROI is shown on the right-hand side. The Gaussian fit through the data points is displayed in turquoise. Scale bar of the ROI: 5 µm.

The CND staining shows an excellent spatial resolution. Dendritic/astrocytic interconnections with diameters of 5–10 pixels, which corresponds roughly to 300–700 nm, and lengths of up to 30 µm are visualized. To measure exemplarily the width of such a fiber (marked with arrows in the ROI in Fig. 7), intensity line plots perpendicular to the fiber direction were analyzed. Since the obtained line plot is a convolution of at least two functions, namely the intensity function of the fiber itself which is determined by the diameter and the (unknown) point spread function of the imaging setup and cannot be clearly defined, a Gaussian fit was chosen to estimate the shape of the lineplot. As a measure for the width, the mean FWHM ± SD of the Gaussian fits of 5 line plots amounts to 0.27 µm ± 0.12 µm (see Fig. 7 for an exemplary fit). This demonstrates that cellular structures with dimensions close to the resolution limit of the imaging setup can be visualized with the CNDs.

Additionally to the CNDs, the organoids were stained with SR101, intended to identify the astrocytes as described above. As seen in Fig. 5B, a rather inhomogeneous staining pattern among the SR101 and the CNDs is observed on both scales (10× and 63× magnification). In some regions, the cells have predominantly taken up CNDs, while the intensities of the SR101 signal is negligible. These could be regions where astrocytes occur less frequently (*e.g.* functional regions with higher abundance of neural stem or progenitor cells). The presence of SR101-free regions is also underlined by the varying SR101 intensity in the 63× magnification images of the different organoid replicates, indicating that different functional areas of the organoid were imaged. In other regions, we observe cells or structures stained by SR101 which show only a weak CND signal (see also Fig. S11). A cell-specific CND number-density could be an explanation for the varied CND intensity. Moreover, it is still to be investigated, whether the specific staining of astrocytes in human iPSC derived organoids can be conducted reliably with SR101 and be implemented as a standard since we would expect high CND signals in all cells, including astrocytes (all living cells of the organoid take up CNDs with a well-defined rate as can be seen from the narrow intensity distribution of the flow cytometry data). Note, however, that cells can be observed as well where SR101 and CNDs are present simultaneously (*e.g.* in image in the bottom right-hand corner).

## 5 Summary and conclusions

We have reported fluorescence and cytometric measurements on brain organoids exposed to carbon nanodots. Our results show that up to organoid diameters of at least a few 100 µm to mm, the living cells within the organoid take up CNDs. This demonstrates that the CNDs have a large penetration depth in the organoid tissue, which we attribute to a combination of their small size, correlating with a high diffusion constant in the extracellular medium, and their relatively slow uptake *via* endocytosis. We have shown that the observed decrease of the fluorescence intensity towards the inner regions of the organoids is due to the extinction coefficient of the excitation and/or the fluorescence light rather than the CND penetration length. In young organoids, the CNDs allow to image typical supercellular structures like rosette formation, while in more mature organoids, the CNDs enable staining of the neuronal network and cellular interconnections.

Flow cytometry shows that in our organoids, most cells are alive. The approaches we studied for the disassembly of the organoids into individual cells, namely by enzymatic and mechanical means, are complementary. While the mechanical disassembly maintains the surface proteins and allows selective labeling of membrane markers, it severely disrupts the cells, leading to a low output of living cells. The enzymatic approach, on the other hand, leaves the cells alive but destroys the surface proteins and with them the option for corresponding staining protocols.

Finally, viability tests show that CNDs localize inside viable cells. Necrotic cells do not collect CNDs since the uptake mechanism is absent, while passive uptake *via* diffusion through a perforated membrane is compensated by washing steps before and after the dissociation of the organoid into individual cells.

Therefore, CNDs show a high capability for staining neuronal networks in brain organoids without affecting the cellular viability. This supports the use of CNDs as fluorescent markers in drug-delivery systems with focus on observing the intracellular and intra-tissue transport and distribution of drugs, especially when applied in neuronal tissue studies.

The versatility of CNDs for brain organoid staining is considered by various factors depending on the intended application and experimental design. Primarily, the observed cell-type independent uptake of pristine CNDs does not allow cell-type-specific staining and cell-type identification. However, whether this can be overcome by surface functionalization, needs to be studied in more detail. Staining with CNDs and simultaneous labeling of different cell-types is further limited due the rarity of cell-type-specific live-cell dyes. Immunofluorescence-based methods may also not be feasible due to the potential leaching of the CNDs during cell permeabilization. Moreover, the emission and excitation properties of the here presented CNDs are in the UV/blue range which limits deep-tissue imaging in comparison to NIR-particles. Also, the influence of the CNDs on the neuronal function of the organoids was not studied. For extended incubation times (>48 h) and/or higher CND concentrations (>0.5 mg ml^−1^), a potential toxicity of the CNDs should be carefully evaluated.

## Author contributions

Data curation, formal analysis, investigation, verification, visualization: CS; project administration, supervision: TH; conceptualization, funding acquisition, methodology, resources: CS, AB, UF, RH and TH; validation, writing: CS, RH and TH.

## Conflicts of interest

There are no conflicts to declare.

## Supplementary Material

RA-016-D6RA01285J-s001

RA-016-D6RA01285J-s002

## Data Availability

Data will be made available upon request. Supplementary information (SI) is available. See DOI: https://doi.org/10.1039/d6ra01285j.
