# Supplementary material for: Incubation of brain organoids with fluorescent carbon nanodots
Source: RSC Adv. 2026 Apr 8;16(21):18745–56. doi: 10.1039/d6ra01285j (PMC13058803; doi:10.1039/d6ra01285j)
Supplement: RA-016-D6RA01285J-s001 [file RA-016-D6RA01285J-s001.pdf]

Supplementary Information (SI) for RSC Advances.
This journal is © The Royal Society of Chemistry 2026

Supplementary Information: Incubation of brain organoids with carbon nanodots

Carla Sprengel,[1] Ute Fischer,[2,3,4] Arndt Borkhardt,[2,3,4] Rainer Haas,[5] and Thomas Heinzel[1,*]

[1] *Condensed Matter Physics Laboratory, Heinrich-Heine-University Düsseldorf, Germany*
[2] *Department of Pediatric Oncology, Hematology and Clinical Immunology, Medical Faculty, Heinrich-Heine-University Düsseldorf, Germany*
[3] *German Cancer Consortium (DKTK), Partner Site Essen/Düsseldorf Düsseldorf, Germany*
[4] *Center for Integrated Oncology Aachen Bonn Cologne Düsseldorf (CIO ABCD), Germany*
[5] *Department of Haematology, Oncology and Clinical Immunology, Medical Faculty, Heinrich-Heine-University Düsseldorf, Germany*

1. CND CHARACTERIZATION TO ENSURE BATCH-TO-BATCH CONSISTENCY

To ensure a batch-to-batch consistency of the CNDs, absorption and fluorescence emission spectra (including the corresponding quantum yield), ^{1}H-NMR spectra and FTIR-spectra of the two batches used in this study were taken. Fig. S1 (S2) shows the data obtained for batch 1 (batch 2). We note that the CND batch 1 was already used for studies in Sprengel et al. [1] and its spectra for a concentration of 8 µg/ml have been published aready.

The absorption spectra of the CNDs from batch 1 (batch 2) show a maximum at 346 nm (347 nm) and a shoulder around 237 nm (237 nm). The fluorescence emission maxima is at 449 nm (452 nm) with a quantum yield of 23.6 % ± 2.3 % (29.5 % ± 3.1 %). These findings are in good accordance with earlier characterization of Kersting et al. [2] and show marginal differences which should not affect the conducted experiments significantly.

The ^{1}H-NMR spectra of batch 1 and 2 show characteristic multipletts between 3.10 ppm and 2.88 ppm and 2.51 ppm and 2.67 ppm. The solvent peak of D_2O was set to 4.79 ppm. A pronounced peak around 2.24 ppm in batch 2 can be explained by residual acetone from the experimental preparation. Due to the naturally occurring inhomogeneity of CNDs in comparison to well-defined molecules, the peaks can be widened and less distinct and were therefore not further analyzed. The spectra had to be recorded with two different spectrometer since the two batches were synthesized at different times, which may also influences the signal-to-noise ratio of the displayed samples.

The displayed FTIR spectra show only marginal differences between the two batches and are in good accordance with the spectra shown by Qu et al. [3], according to whom the peaks between 2857 cm^{-1} and 3059 cm^{-1} arise from sp^2 and sp^3 C-H bonds. The bond around 3264 cm^{-1} can be attributed to N-H, while the peaks between 1000 cm^{-1} and 1650 cm^{-1} have been tentatively attributed to C=O, COO$^-$, COO, C-N and C-OH vibtrations.

CND Batch 1

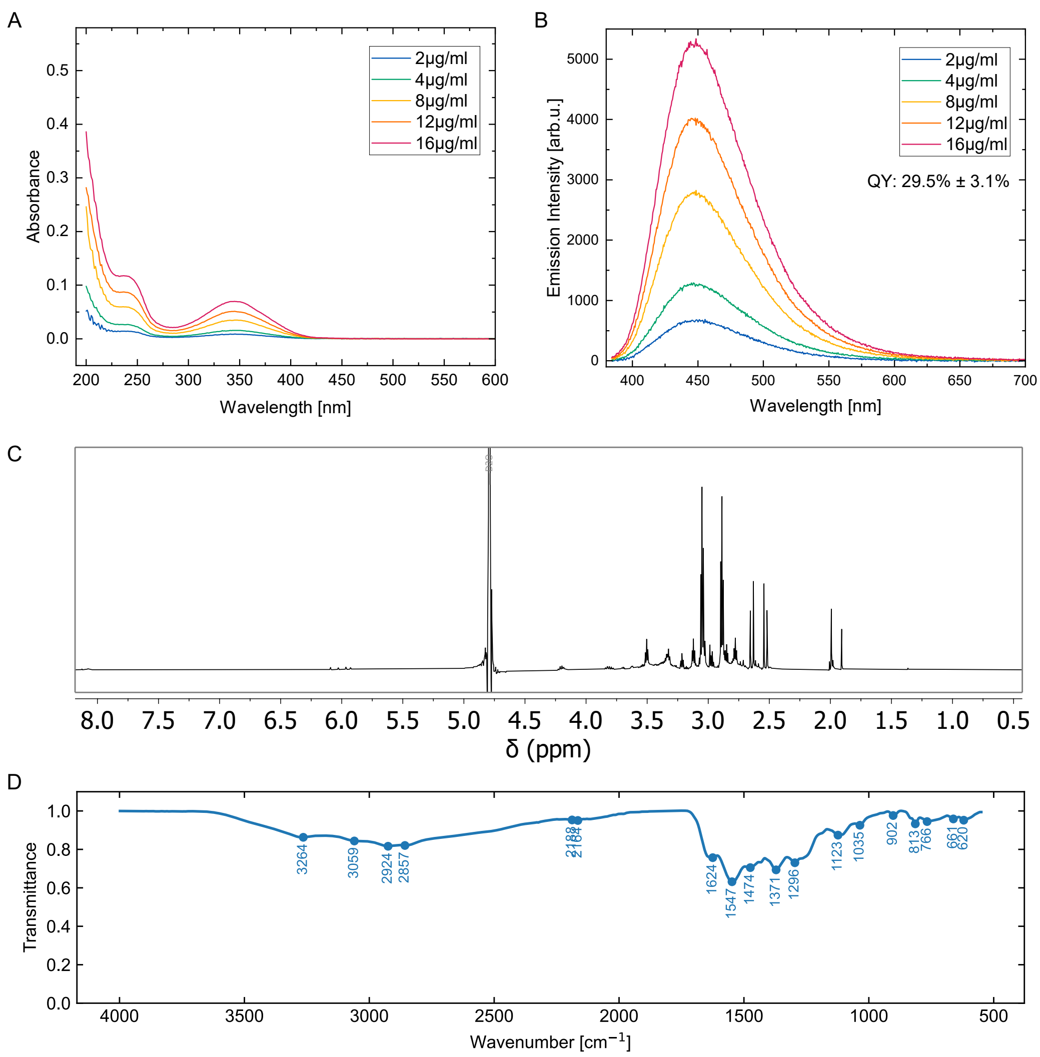

FIG. S1. Analysis conducted with the CNDs used in this work as a quality control to ensure batch-to-batch consistency. Here, the first Batch of CNDs of this study is presented. Absorption spectra (A) and fluorescence emission spectra (B) after excitation at 360 nm are displayed for different CND concentrations with their corresponding relative quantum yield (QY) as well as the according ^{1}H-NMR spectrum (C) and the FTIR-spectrum (D). The solvent peak of D_2O in the ^{1}H-NMR spectra amounts to 4.79 ppm. Please note, that the CND Batch 1 was already used for studies in Sprengel et al. [1] and therefore, the absorption and emission spectra of the 8 μg/ml concentration as well as the ^{1}H-NMR have been published there already.

CND Batch 2

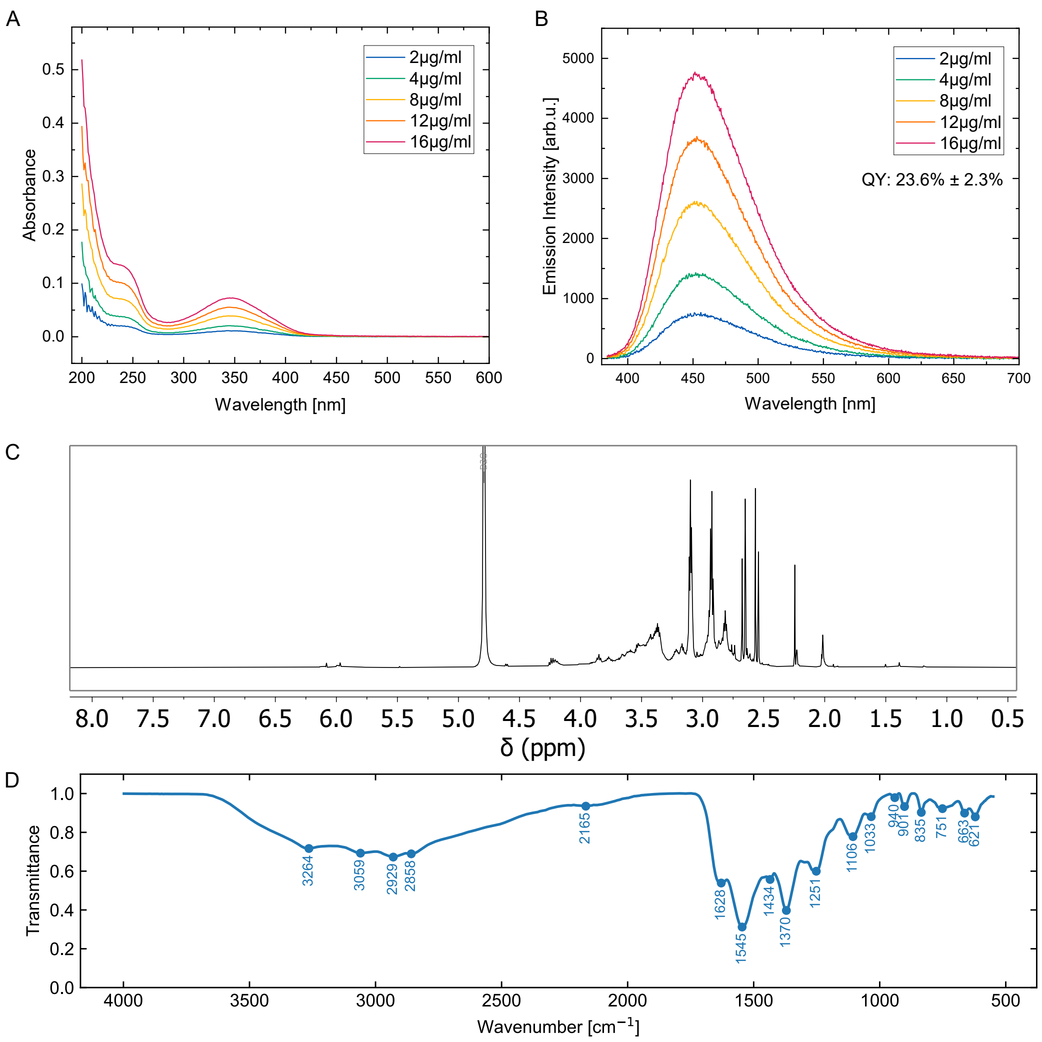

FIG. S2. Analysis conducted with the CNDs used in this work as a quality control to ensure batch-to-batch consistency. Here, the second Batch of CNDs of this study is presented. Absorption spectra (A) and fluorescence emission spectra (B) after excitation at 360 nm are displayed for different CND concentrations with their corresponding relative quantum yield as well as the according ^{1}H-NMR spectrum (C) and the FTIR-spectrum (D). The solvent peak of D_2O in the ^{1}H-NMR spectra amounts to 4.79 ppm.

2. ADDITIONAL SCATTER PLOTS REGARDING THE 7AAD STAINING

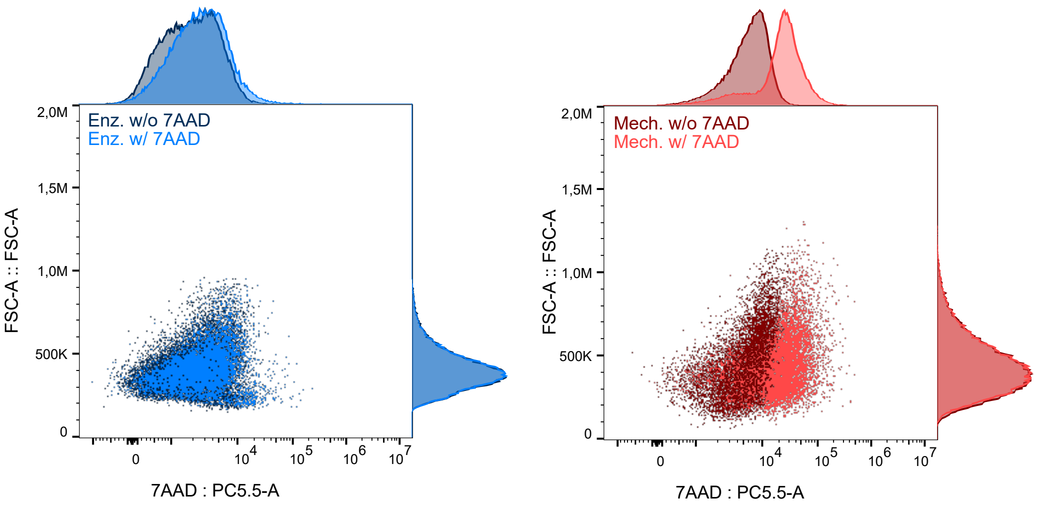

FIG. S3. Comparison of the enzymatic and mechanical dissociation of organoids based on flow cytometry analysis. Enzymatic dissociation was performed as described in the methods section. For the Mechanical dissociation, the organoids were directly mechanically triturated by pipetting after washing without an incubation in the enzyme solution. The further processing of the cell suspension was carried out analogously for both methods as described in the *Methods* section. The comparison of cells from enzymatically and mechanically dissociated organoids (EDOs respective MDOs) that were stained with or without 7AAD was performed to ensure a distinction of the 7AAD fluorescence and cellular autofluorescence. For EDOs, an enhanced 7AAD signal is seen for a few cells in comparison to the unstained control. For MDOs, the 7AAD uptake is clearly distinguishable from the autofluorescence signals of the cells. It can thus be concluded that the staining with 7AAD for live/dead cells was successful.

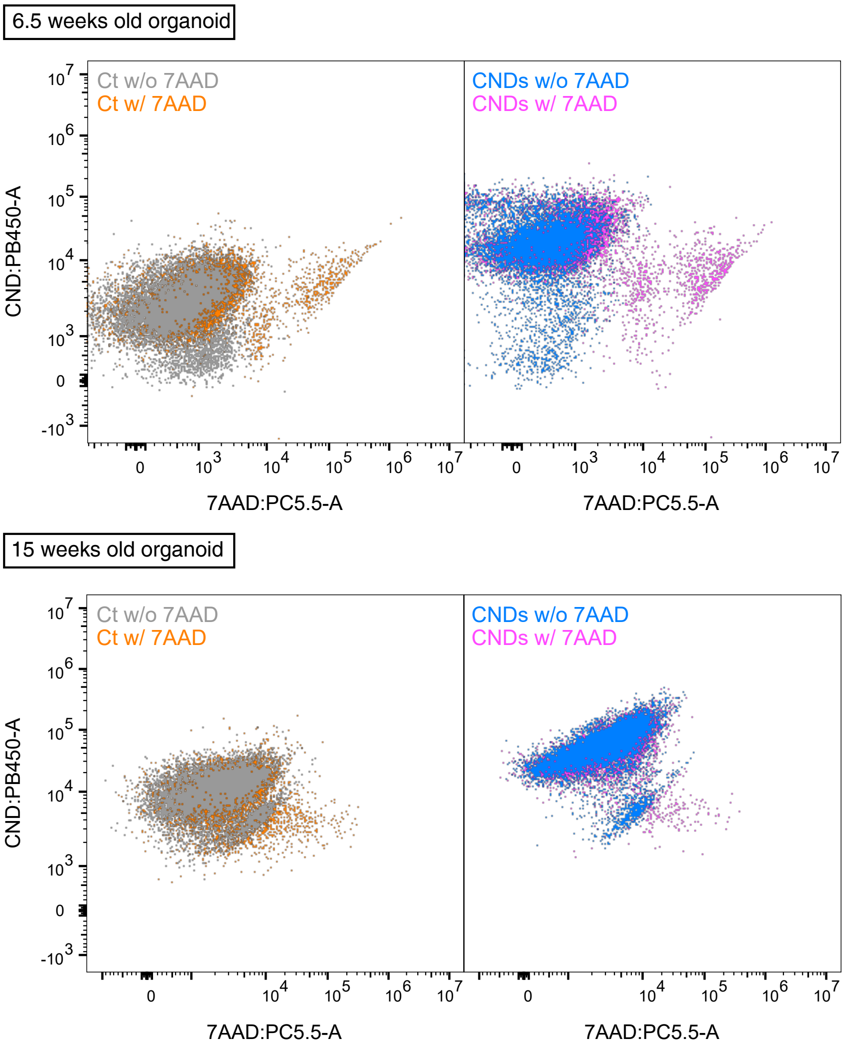

FIG. S4. Exemplary scatter plots of the CND:7AAD signals of the 6.5 weeks old and the 15 weeks old organoids (gated for single cells) that were incubated with or without CNDs ("CND" and "Ct" samples) and with or without 7AAD (w/ 7AAD and w/o 7AAD). Uptake of the 7AAD marker in dead cells is seen by the shift of the fluorescence intensity of those cells in the 7AAD (PC5.5) channel when comparing the w/o to the w/ 7AAD samples. Uptake of CNDs is only visible for 7AAD$^-$ populations.

3. METABOLIC FUNCTION ANALYSIS BASED ON JC-1 STAINING

In Figure S5, the analysis of the viability based on 7AAD or JC-1 staining of 8 week old organoids after enzymatic or mechanical dissociation and CND or FCCP treatment is displayed. As seen from the 7AAD signal, mechanical dissociation of the organoids leads to a poor viability of the cells (accords to the loss of cell membrane integrity) in comparison to the enzymatically dissociated organoids as was also seen in the 15 week old organoids (Fig. 2 in main text). Since the 7AAD uptake is not changed biologically relevant after CND or FCCP incubation in comparison to the control, no influence on the integrity of the cell membranes could be detected for those conditions.

To investigate the metabolic activity as a measure for the viability, the widely used JC-1 staining was performed. It is used to analyze the influence of the treatments on the mitochondrial membrane potential $\Delta\Psi_m$ of the cells [4, 5]. JC-1 shows green fluorescence emission in its monomeric form which is shifted towards higher wavelength when JC-1 aggregates. In healthy cells, $\Delta\Psi_m$ causes an influx of the cationic JC-1 monomers into the mitochondria, leading to the aggregation of the JC-1 monomers which can be detected by a higher fluorescence emission in the yellow to red fluorescence channel (like the PE channel). At low $\Delta\Psi_m$ as seen in apoptotic cells, JC-1 monomers do not form aggregates which is reflected as a high intensity in the green fluorescence channel (e.g. the FITC channel). Based on this, the ratio of JC-1 monomer$^-$ and JC-1 monomer$^+$ events can be used as a measure for the relative mitochondrial activity.

Usually, a low intensity in the yellow-orange PE channel should occur with high fluorescence intensity in the green channel, which is not as pronounced in the measured samples as expected from theory. Since the fluorescence in the samples treated with the depolarizer FCCP is shifted towards high intensities in the FITC channel, the ratio of JC-1 monomer$^-$ (JC-1m$^-$) and JC-1 monomer$^+$ (JC-1m$^+$) events was still used as a measure for mitochondrial activity. Since JC-1 is poorly water soluble, precipitated JC-1 aggregates in the media or on the cell surfaces may cause additional fluorescence in the red channel that is unrelated to the cellular viability. The results of the ratio of events gated by the marked JC-1m$^\pm$ regions in Fig. S5 is displayed in Table I. Apparently, incubation with CNDs does not influence $\Delta\Psi_m$ significantly in comparison to the control samples. A ratio of $r \equiv \frac{JC-1m^-}{JC-1m^+} > 1$ means that there are more viable cells present than cells with a low metabolic activity. Mechanical dissociation, on the other hand, leads to a significant decrease in metabolic activity, with $r = 0.13 \pm 0.002$ which correlates with a low $\Delta\Psi_m$. Therefore the 7AAD- and JC-1 stainings indicate that mechanical dissociation decreases the cell viability significantly since the cell membrane integrity is damaged (7AAD staining) and metabolic activity of the cells is influenced negatively (JC-1 staining).

TABLE I. Ratio $r = JC - 1m^-/JC - 1m^+ \pm SD$ of the events in the JC-1m$^-$ and JC-1m$^+$ marked regions in Fig. S5 for the samples (1)-(3). The statistical significance (sign.) was analyzed with an independent two-sample t-test with Welch's correction with a significance level of $\alpha = 0.05$ in comparison to the control (1). The corresponding p values are displayed accordingly. 4 organoids were pooled and measured in 3 technical replicates per sample.

Sample	$r\pm$ SD	sign., $\alpha = 0.05$	p value
(1) Ct Enz.	2.6 ± 0.1	-	-
(2) Mech	0.13 ± 0.002	sign.	$3.4 \cdot 10^{-4}$
(3) CNDs Enz.	2.7 ± 0.3	Not sign.	0.94

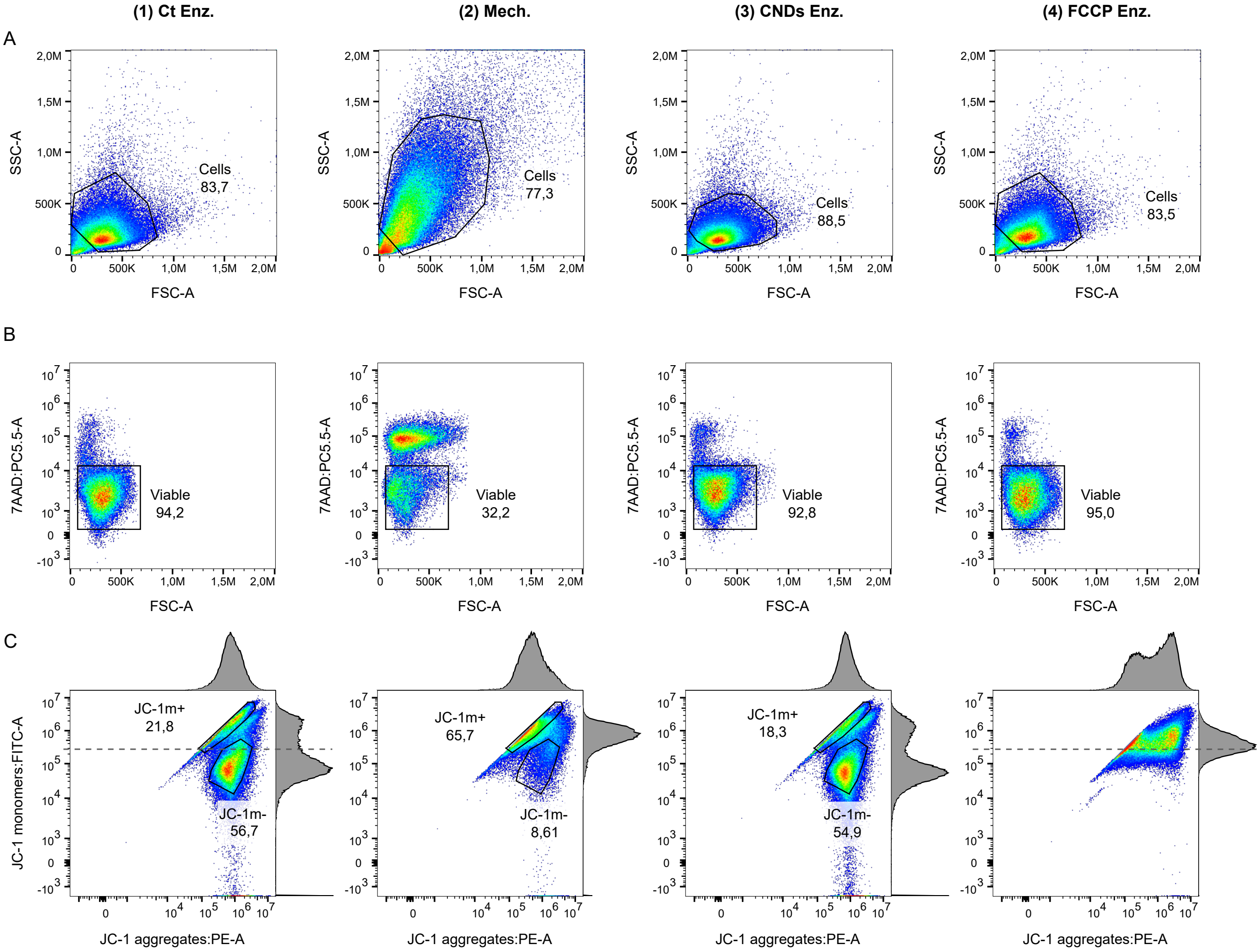

FIG. S5. Analysis of the viability of 8 week old organoids based on 7AAD or JC-1 staining. Displayed are 4 different samples: (1) enzymatically dissociated organoids labelled as *Ct Enz.*, (2) mechanically dissociated organoids (*Mech.*), (3) Organoids that were incubated with CNDs for 48 h and enzymatically dissociated (*CNDs Enz.*) and (4) Organoids that were enzymatically dissociated and treated with 100 µM FCCP afterwards (*FCCP Enz.*). Scatter plots were obtained from flow cytometry. In row A, the SSC:FSC scatter plots of the samples are displayed. Scatter plots to determine the viability based on the 7AAD staining (PC5.5 channel) are shown in row B. To investigate the relative influence of the treatments on the mitochondrial membrane potential $\Delta\Psi_m$ of the samples, the scatter plots of the signal of JC-1 monomers (FITC channel) versus JC-1 aggregates (PE channel) are displayed in row C. FCCP was used to depolarize the $\Delta\Psi_m$. The dashed line in plot (1)C and (4)C corresponds to the same intensity in the FITC channel. 4 organoids were pooled and measured in 3 technical replicates per sample.

4. ADDITIONAL MICROSCOPY IMAGES OF NEUROSPHERES

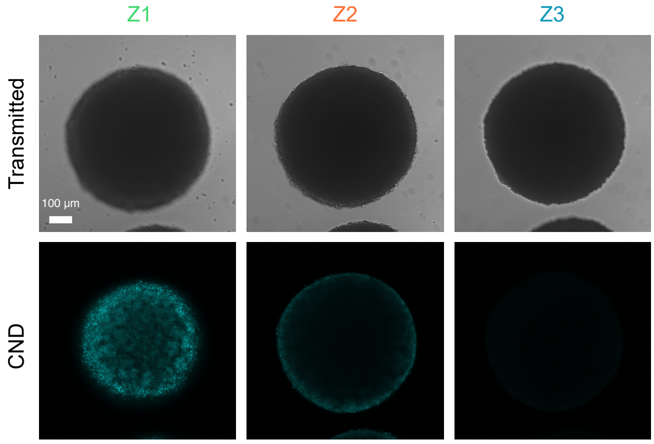

FIG. S6. Fluorescent microscopy images of neurospheres at day 9 in culture after a CND incubation for 48 h. The displayed planes z1, z2 and z3 correspond to the planes discussed in the main text (see Fig. 4). Plane z1 and z3 were recorded symmetrically around plane z2 with a distance to plane z2 of 102 µm. The scale bar of 100 µm applies to all images.

5. Z-STACK ANALYSIS OF 28 DAY OLD ORGANOIDS STAINED WITH CNDS

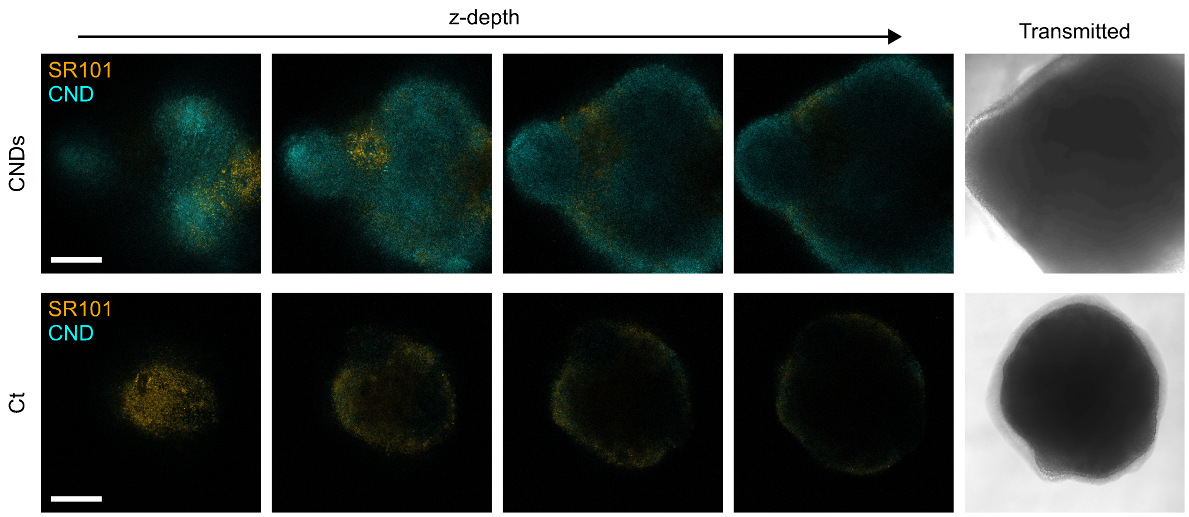

FIG. S7. Confocal fluorescence microscopy images of different confocal slices of organoids at day 28 of culture that were incubated with or without CNDs for 48h are shown. SR101 was used to stain the astrocytes. It is observed that the CNDs can be detected even tens of micrometers inside the organoids which accords to multiple cell-layers that were penetrated by the CNDs. Neural rosettes are no longer present indicating maturation of the organoids. At higher imaging depths the signals get attenuated due to the limitation of the imaging setup (light penetration into tissue and working distance of the imaging setup). Distance between confocal planes is 31µm. Scale bar: 200µm.

A quantitative analysis of the fluorescence intensity decay across different imaging depths of 28 days old organoids is displayed in Fig. S8. For a control organoid (no CND incubation, only autofluorescence is present) and a CND-organoid (incubation for 48 h with CNDs), n=5 ROIs each were chosen randomly and kept constant across all detected z-planes (up to 90 µm into the sample).

For each ROI, the mean fluorescence intensity was calculated in every z-plane and is shown vs. the corresponding imaging depth in Fig. S8 A for one ROI from each organoid and in Fig. S8 B for the five ROIs from each organoid. Two representative microscopy images at an z-depth of 15.3 µm are displayed with the 5 ROIs marked in Fig. S8C. The autofluorescence in the control organoid shows a similar spatial decay as the fluorescence intensity of the organoid exposed to CNDs. The half-value depth HVD for the intensity decay is obtained from exponential fits of the data points subsequent to the signal maximum. The penetration depths amount to 36.0 µm $\pm$ 3.5 µm for the autofluorescence of the control organoids and 34.5 µm $\pm$ 2.7 µm for the CND-organoid, i.e., they are equal within the standard error. Hence, the attenuation is dominated by light-tissue interaction while the finite penetration depth of the CNDs into the organoid is negligible.

The detected mean intensity does not peak at an imaging depth of 0 µm for all ROIs in both organoids. This is most likely explained by artifacts occurring during the measurement and the ROI analysis. As the images were obtained by confocal microscopy, the detected plane is limited to a few µm defined by the confocal volume. The experimentally defined surface of the organoid (at 0 µm) was set at the point where a signal from the organoid was detected the first time. At this point, the confocal volume was most likely not fully submerged into the sample resulting in lower detected intensities. Additionally, the position of the ROIs relative to the organoid may influence the depth of the intensity maxima. Not all ROIs coincide with *inner* regions of the organoid, that have their surface at an imaging depth of 0 µm. In more *peripheral* regions, the organoid surface appears at higher depths, resulting in a shift of the intensity maxima towards higher imaging depths.

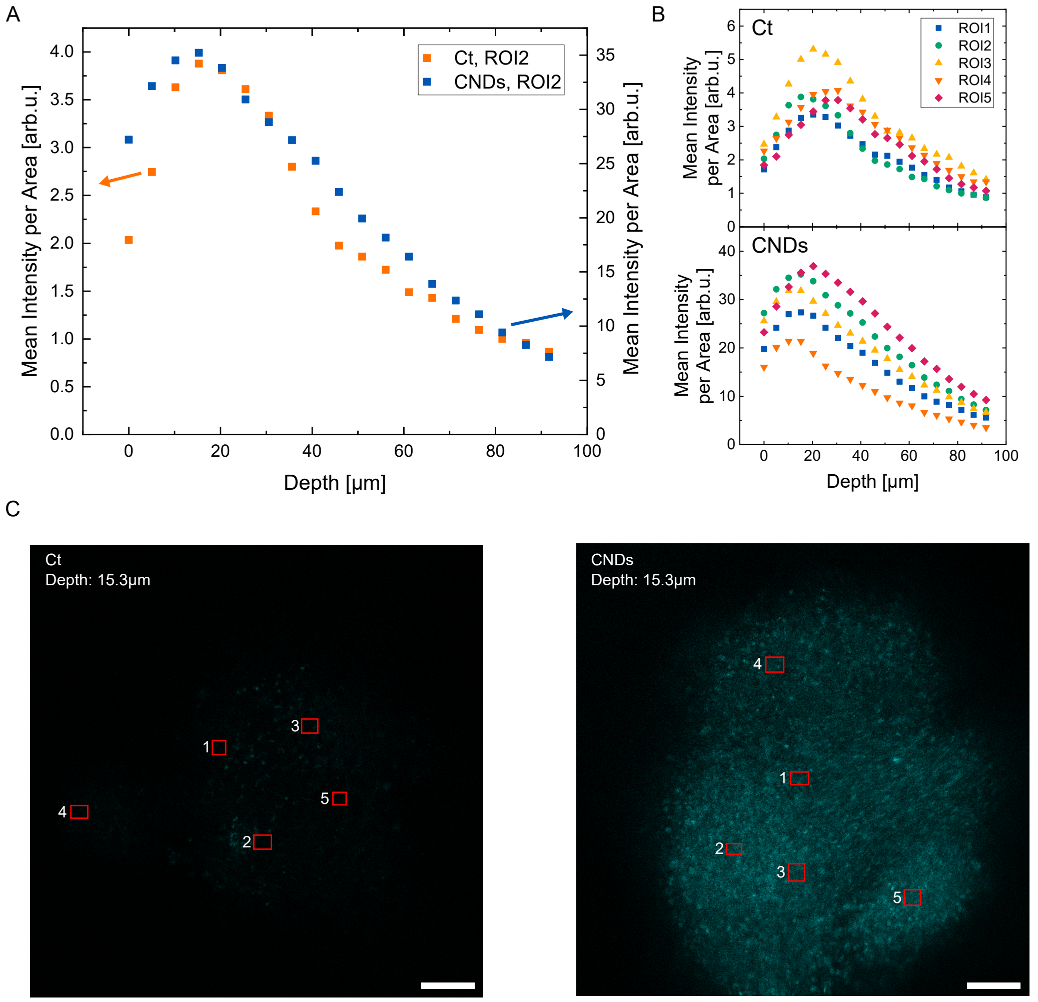

FIG. S8. Quantitative analysis of the detected fluorescence intensity as a function of imaging depth for a control organoid (Ct) and a CNDs-treated organoid (CNDs). A direct comparison of the intensity decay for a representative ROI of each sample is shown in A. In B, the intensity as a function of the depth of 5 randomly selected ROIs (including the ROIs displayed in A) for each organoid is displayed. The corresponding ROIs are marked in the confocal microscopy images of the CND channel of each organoid at the imaging depth of 15.3 μm in C. The ROIs were kept constant across all z-planes. Scale bars: 100μm.

6. ENDOCYTOTIC UPTAKE OF CNDS INTO MCF-7 CELLS

To investigate whether endocytosis is the primary CND uptake mechanism into cells (as expected from the results in the main Section 4.5), a 4 °C test was performed using MCF-7 cells. As incubation at 4 °C is commonly used to suppress endocytosis [6, 7], the CND uptake into cells is expected to be marginal at those temperatures compared to the uptake at 37 °C. MCF-7 cells were incubated with and without CNDs at 37 °C and at 4 °C for 2 h and 4 h. As shown in Fig. S9, incubation with CNDs at 37 °C leads to higher fluorescence signals in the CND channel compared to the cellular autofluorescence while the incubation with CNDs at 4 °C does not alter the signal intensities. The uptake of CNDs at low temperature is thus marginal and therefore suggests that CNDs are primarily taken up via endocytosis.

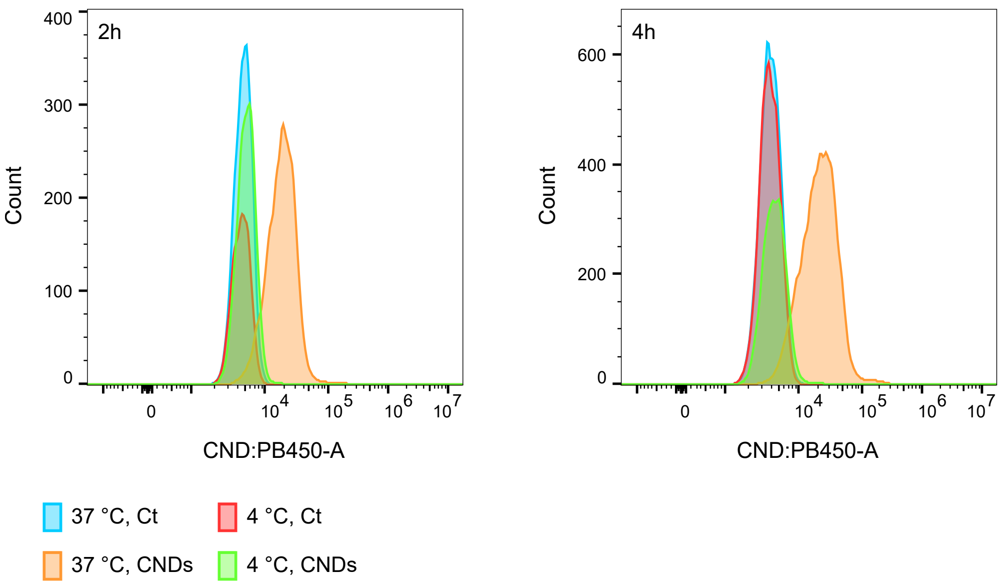

FIG. S9. Uptake of CNDs into MCF-7 cells at 4 °C and 37 °C. MCF-7 cells were incubated with CNDs ("CNDs") and without CNDs ("Ct") at 4 °C and 37 °C for 2 h and 4 h. Displayed are the intensity distributions in the CND channel after flow cytometry measurement of the samples.

Method: MCF-7 cells were cultivated in RPMI 1640 (Biowest, #L0501) supplemented with 10 % FBS (Sigma, #F2442), 100 U ml^{-1} penicillin and 100 µg ml^{-1} streptomycin (Sigma, #P0781) and 300 mg ml^{-1} L-Glutamin (Sigma, #G7513) in an incubator with humidified air at 37 °C and 5% CO_2. The cells were passaged using a Trypsin-EDTA solution (Sigma, #T3924) every 3 to 5 days. For the 4 °C assay, the cells were incubated for 30 min at 4 °C before adding 0.5 mg ml^{-1} CNDs or the equal volume of DPBS as a solvent control. The cells were incubated for another 2 h or 4 h at 4°C. Control samples were prepared accordingly with all incubation steps performed at 37 °C. The samples were washed twice with DPBS before harvesting was performed using the Trypsin-EDTA solution. The enzymatic reaction was stopped by resuspending the samples in culture medium. After harvesting, the cells were centrifuged, solved in FACS buffer and filtered using 40 µm cell strainer. Flow cytometry measurement was further conducted using a CytoFlex cytometer (Beckman Coulter) with the CND detection in the PB450 channel (ex. 405 nm, em. 450 nm/45 nm BP). The data analysis was performed using the FlowJo™ v10.10.0 Software (BD Life Sciences).

7. ADDITIONAL MICROSCOPY IMAGES WITH LYSOTRACKER OR SR101 STAINING

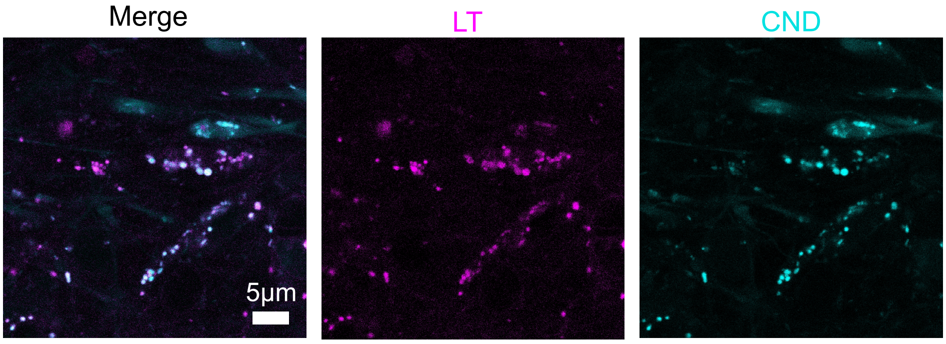

FIG. S10. Confocal fluorescent images of an representative organoid at day 28 of culture after CND incubation. Lysosomes were stained with Lysotracker ("LT") and are displayed in magenta. The CND channel is shown in cyan. In the merged image, overlay of CNDs and Lysotracker signals can be observed, indicating that CNDs accumulate inside the lysosomes. The two fluorescent channels were imaged simultaneously. A timeseries of the displayed organoid is presented as a supplementary video.

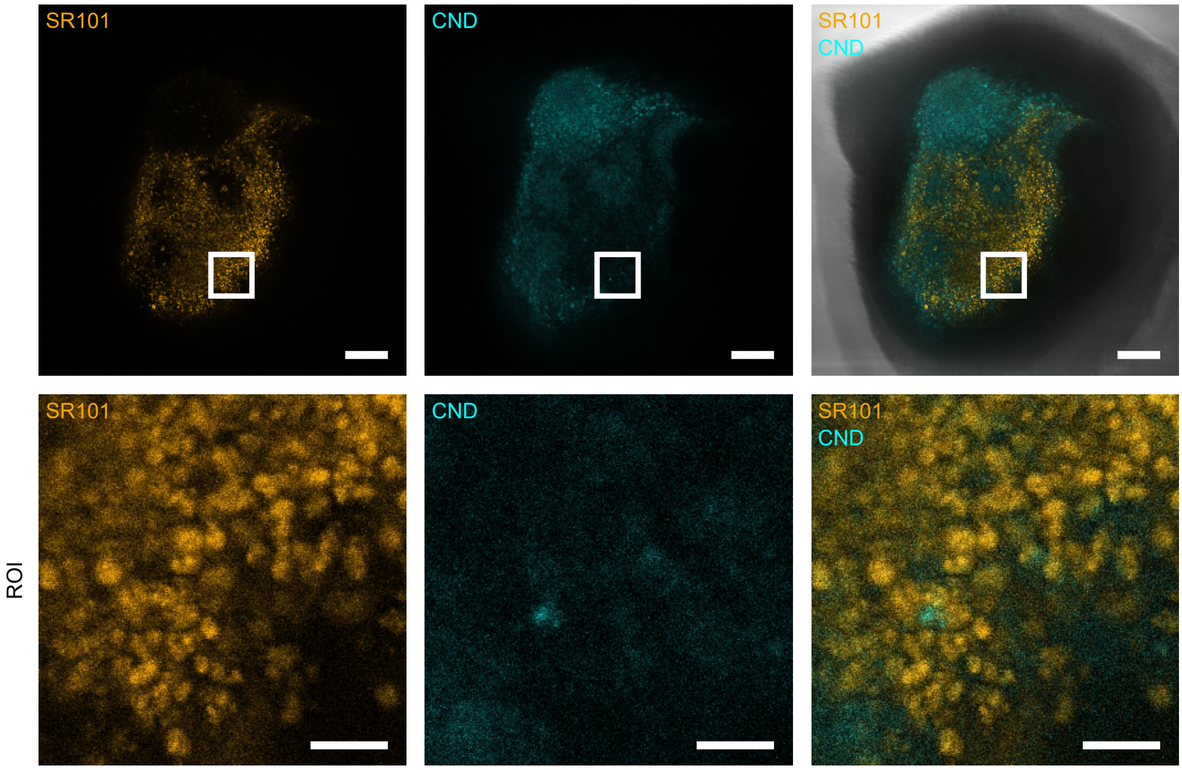

FIG. S11. Typical confocal microscopy images of an organoid at day 28 of culture after CND incubation. SR101 was used to stain the astrocytes. The individual images of the SR101 and the CND channel are shown alongside the overlay with the transmission picture in the first row. In the second row, the zoom into the marked region of interest (ROI) is depicted. An area with almost exclusive SR101 fluorescence and weak CND fluorescence is observed here. Scale bar: 100µm, ROIs: 20µm.

* thomas.heinzel@hhu.de

[1] C. Sprengel, C. David, L. Berning, C. Nollmann, T. Lenz, K. Stühler, B. Stork, and T. Heinzel, Lysosomal activity in response to the incubation of pristine and functionalized carbon nanodots, iScience **28**, 111654 (2025).

[2] D. Kersting, S. Fasbender, R. Pilch, J. Kurth, A. Franken, M. Ludescher, J. Naskou, A. Hallenberger, C. v. Gall, C. J. Mohr, R. Lukowski, K. Raba, S. Jaschinski, I. Esposito, J. C. Fischer, T. Fehm, D. Niederacher, H. Neubauer, and T. Heinzel, From in vitro to ex vivo: subcellular localization and uptake of graphene quantum dots into solid tumors, Nanotechnology **30**, 395101 (2019).

[3] D. Qu, M. Zheng, J. Li, Z. Xie, and Z. Sun, Tailoring color emissions from n-doped graphene quantum dots for bioimaging applications, Light: Science and Appl. **4**, e364 (2015).

[4] F. Sivandzade, A. Bhalerao, and L. Cucullo, Analysis of the mitochondrial membrane potential using the cationic jc-1 dye as a sensitive fluorescent probe, Bio-protocol **9**, e3128 (2019).

[5] S. T. Smiley, M. Reers, C. Mottola-Hartshorn, M. Lin, A. Chen, T. W. Smith, G. D. Steele, and L. B. Chen, Intracellular heterogeneity in mitochondrial membrane potentials revealed by a j-aggregate-forming lipophilic cation jc-1., Proc. Natl. Acad. Sci. USA **88**, 3671 (1991).

[6] J. J. Rennick, A. P. R. Johnston, and R. G. Parton, Key principles and methods for studying the endocytosis of biological and nanoparticle therapeutics, Nat. Nanotechnol. **16**, 266 (2021).

[7] S. C. Silverstein, R. M. Steinman, and Z. A. Cohn, Endocytosis, Ann. Rev. Biochem. **46**, 669 (1977).
